# Vaccination with an mRNA-encoded membrane-bound HIV Envelope trimer induces neutralizing antibodies in animal models

**DOI:** 10.1126/scitranslmed.adw0721

**Published:** 2025-07-30

**Authors:** Parham Ramezani-Rad, Christopher A. Cottrell, Ester Marina-Zárate, Alessia Liguori, Elise Landais, Jonathan L. Torres, Amber Myers, Jeong Hyun Lee, Sabyasachi Baboo, Claudia Flynn, Katherine McKenney, Eugenia Salcedo, Xiaoya Zhou, Oleksandr Kalyuzhniy, Erik Georgeson, Nicole Phelps, Danny Lu, Saman Eskandarzadeh, Sergey Menis, Michael Kubitz, Bettina Groschel, Nushin Alavi, Abigail M. Jackson, Wen-Hsin Lee, Andy S. Tran, Elana Ben-Akiva, Katarzyna Kaczmarek Michaels, Jolene K. Diedrich, Chiamaka A. Enemuo, Vanessa Lewis, Arpan Pradhan, Sudhir Pai Kasturi, Torben Schiffner, Jon M. Steichen, Diane G. Carnathan, Sunny Himansu, John R. Yates, James C. Paulson, Gabriel Ozorowski, Darrell J. Irvine, Guido Silvestri, Devin Sok, Andrew B. Ward, Shane Crotty, William R. Schief

**Affiliations:** 1 Center for Vaccine Innovation, La Jolla Institute for Immunology, La Jolla, CA 92037, USA; 2 Consortium for HIV/AIDS Vaccine Development (CHAVD), The Scripps Research Institute, La Jolla, CA 92037, USA; 3 Department of Immunology and Microbiology, The Scripps Research Institute, La Jolla, CA 92037, USA; 4 IAVI Neutralizing Antibody Center, San Diego, CA 92121, USA; 5 Department of Integrative Structural and Computational Biology, The Scripps Research Institute, La Jolla, CA 92037, USA; 6 Department of Molecular Medicine, The Scripps Research Institute, La Jolla, CA 92037, USA.; 7 Department of Biological Engineering, Massachusetts Institute of Technology, Cambridge, MA 02139, USA; 8 Emory National Primate Research Center and Emory Vaccine Center, Emory University School of Medicine, Atlanta, GA 30329, USA; 9 Moderna, Inc. Cambridge, MA 02142, USA; 10 Howard Hughes Medical Institute, Chevy Chase, MD 20815, USA; 11 Department of Medicine, Division of Infectious Diseases and Global Public Health, University of California, San Diego (UCSD), La Jolla, CA 92037, USA

## Abstract

A protective vaccine against human immunodeficiency virus (HIV) will likely need to induce broadly neutralizing antibodies (bnAbs) that engage relatively conserved epitopes on the HIV envelope glycoprotein (Env) trimer. Nearly all vaccine strategies to induce bnAbs require the use of complex immunization regimens involving a series of different immunogens, most of which are Env trimers. Producing protein-based clinical material to evaluate such relatively complex regimens in humans presents major challenges in cost and time. Furthermore, immunization with HIV trimers as soluble proteins induces strong non-neutralizing responses to the trimer base, which is normally occluded on the virion. These base responses could potentially detract from the elicitation of nAbs and the eventual induction of bnAbs. mRNA vaccine platforms offer potential advantages over protein delivery for HIV vaccine development, including increased production speed, reduced cost, and the ability to deliver membrane-bound trimers that might facilitate improved immuno-focusing to non-base epitopes. We report the design of mRNA-delivered soluble and membrane-bound forms of a stabilized native-like Env trimer (BG505 MD39.3), initial immunogenicity evaluation in rabbits that triggered clinical evaluation, and more comprehensive evaluation of B cell, T cell, and antibody responses in non-human primates. mRNA-encoded membrane-bound Env immunization elicited reduced off-target base-directed Env responses and stronger nAb responses compared with mRNA-encoded soluble Env. Overall, mRNA delivery of membrane-bound Env appears promising for enhancing B cell responses to subdominant epitopes and facilitating rapid translation to clinical testing, which should assist HIV vaccine development.

## Introduction

Effective vaccine strategies are required to build immunity to pathogens with substantial global health threats. The human immunodeficiency virus (HIV) pandemic has been recognized since 1981, but HIV continues to infect and kill millions of people annually (1). HIV represents a remarkable vaccine challenge due to its genetic diversity, immune escape, and integration into the host genome (2). The envelope glycoprotein (Env) trimer on the surface of HIV is the sole target of neutralizing antibodies (nAbs), and nAbs to different Env epitopes can confer protective responses to varying numbers of HIV strains (3). However, nAbs are difficult to elicit due to the immunodominance of non-neutralizing epitopes on Env immunogens and the abundant glycosylation of the Env trimer, which limits accessibility to neutralizing epitopes. Owing to the antigenic diversity of HIV Env across different isolates, most vaccine design strategies aim to induce broadly neutralizing antibodies (bnAbs), defined as nAbs that neutralize diverse HIV isolates (2). Inducing bnAbs faces additional challenges, including the low frequency of naive bnAb precursor B cells with the genetic and structural potential to develop into bnAbs and the need to guide somatic hypermutation (SHM) to produce bnAbs. Nearly all bnAb vaccine strategies currently being pursued involve sequential immunization with different immunogens, and most immunogens are envisaged to be based on stabilized prefusion HIV Env trimers (4–7). This strategy is thought to be necessary both to focus responses to bnAb epitopes and to elicit the SHM needed for bnAb activity.

The advent of the mRNA vaccines against coronavirus disease 2019 (COVID-19) introduced a rapid, effective, and promising vaccine platform for human use (8). To optimally utilize mRNA for HIV vaccine development, it will be important to evaluate different strategies to deliver HIV Env trimers by mRNA-lipid nanoparticle (LNP). Compared with protein vaccines, mRNA vaccines translate the immunogen protein directly in transfected cells in vivo, which enables new modes of antigen delivery, such as membrane-bound antigen delivery, but also increases the burden on antigen design to develop antigens that fold and assemble properly without the benefit of purification. Purified soluble protein trimers employed in many HIV vaccine studies expose an extensive non-glycosylated, non-neutralizing, and typically highly immunogenic surface at the trimer base that is normally occluded on virion-associated, membrane-bound HIV Env (9). In contrast, mRNA delivery of membrane-bound Env trimer has the potential to focus responses to non-base epitopes required for bnAb development. mRNA delivery of membrane-bound HIV Env trimers engineered as germline-targeting priming or first-boosting immunogens has proven effective in mouse models (10–12). mRNA delivery of native-like HIV Env trimers has been shown to induce autologous nAb responses in rabbits (13) and non-human primates (NHPs) (14, 15). Here, we describe the design and rabbit immunogenicity evaluation of mRNA-delivered soluble and membrane-bound HIV Env native-like BG505-isolate-derived MD39.3 trimers that led to their clinical evaluation in HVTN302 (NCT05217641) (16, 17). We then evaluate NHP B cell, T cell, and antibody responses to mRNA-delivered soluble and membrane-bound MD39.3 trimers compared with a matched MD39.3 soluble protein trimer control with a potent adjuvant.

## Results

### MD39.3 mRNA immunogens were developed and characterized in vitro

**Fig. 1.**
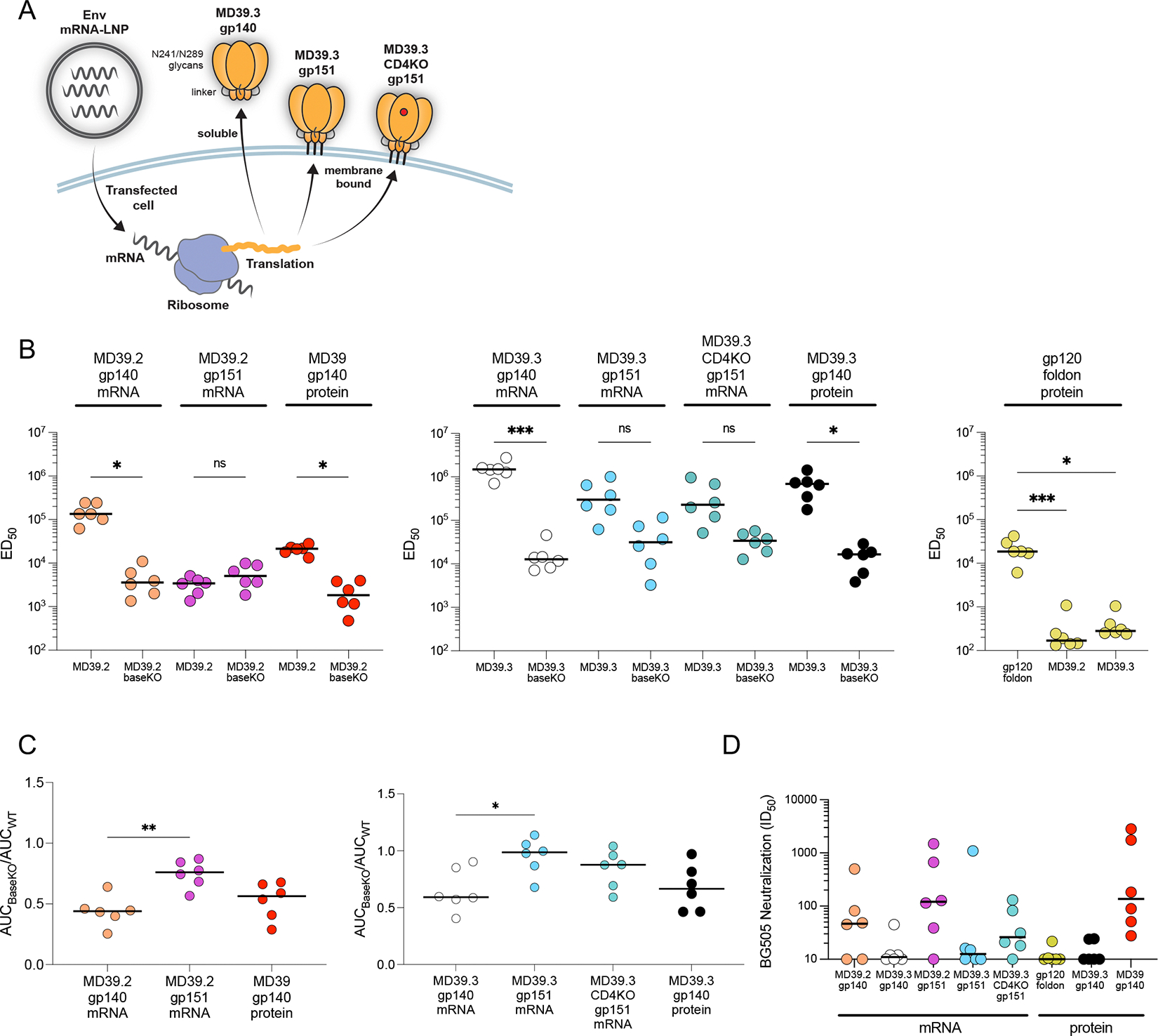
Membrane-bound Env mRNA elicited lower base-directed antibody responses than soluble Env in rabbits. (**A**) Illustration of MD39.3 mRNA immunogen expression. The cartoon illustrates the in vivo translation of MD39.3 mRNA in transfected cells, producing either soluble or membrane-bound MD39.3 proteins. All MD39.2 and MD39.3 variants are designed with furin cleavage independence provided by a Link14 linker. MD39.3 has a filled glycan hole achieved through N241/N289 glycans. The red circle denotes a mutation in the CD4 binding site to block binding to CD4. (**B**) ED_50_ values for serum antibodies binding to matched parental antigens or their BaseKO versions were measured by ELISA. Sera from the gp120 foldon control group were tested against matched antigen and MD39.2 or MD39.3 antigens. Statistical comparisons were performed within each group between antigens. (**C**) Shown are the ratios of AUC values for BaseKO antigen over AUC values for parental antigen. Lower values indicate the presence of more base binding antibodies in the sera. Statistical comparisons were performed between soluble and membrane-bound forms of MD39.2 and MD39.3. (**D**) Serum neutralization against BG505 T332N pseudovirus is reported as ID_50_ values. Statistical comparisons were performed between soluble and membrane-bound forms of MD39.2 and MD39.3. Bars indicate median values for ED_50_, AUC measurements, and neutralization data. Each point indicates a single animal (n=6 per group). Statistical significance was assessed using the Mann-Whitney test or Kruskal-Wallis test, followed by Dunn’s multiple comparisons test. Significance levels are indicated as ns *P* > 0.05, *P < 0.05, and ***P < 0.001.

For mRNA delivery of BG505 MD39 trimers, we designed soluble gp140 and membrane-bound gp151 forms that contained the furin cleavage-independent linker, Link14, between gp120 and gp41 (18), which we refer to as MD39.2, or contained the Link14 linker and “congly” mutations to restore the glycosylation sites at positions 241 and 289 (19), which we refer to as MD39.3 (Fig. 1A). The gp140 versions were truncated at residue 664, as was originally done for BG505 SOSIP (20) and later for BG505 MD39 (21). For the gp140 constructs, the antigenic profiles assessed by biolayer interferometry (BLI), expression yields, thermal stabilities, predominance of native-like conformations as assessed by negative stain electron microscopy (nsEM), and glycosylation profiles were similar to those for the original cleaved BG505 MD39 gp140 trimer (21) (fig. S1A to D). BG505 MD39 and BG505 MD39.3 both bound to human CD4 but did not undergo the conformational change necessary to expose the CD4-induced epitope of 17b (fig. S1E and F). Additionally, a variant of BG505 MD39.3 containing the G473T mutation to prevent the trimer from binding to human CD4 (CD4KO) (19), which could potentially affect its biodistribution, was produced and characterized (fig. S1F). The antigenic profiles for the gp151 versions showed appreciable binding to several bnAbs and reduced or undetectable binding to non-nAbs, similar to a cell-surface MD39 gp160 positive control and in contrast with a cell-surface negative control gp120 trimer (fig. S2A and B).

### Membrane-bound MD39.3 elicited nAbs with reduced base-directed antibodies in rabbits

For initial immunogenicity evaluation, we carried out a rabbit study (table S1) in which animals were immunized intramuscularly in the right hind thigh with either 100 εg mRNA-LNPs or 30 εg protein plus 375 εg SMNP adjuvant (22) at weeks 0, 8, and 24. All vaccines were based on the BG505 isolate. We tested mRNA-encoded MD39.2 and MD39.3 as both gp140 and gp151, plus the CD4KO variant of MD39.3 gp151. Adjuvanted protein vaccine controls included MD39.3 gp140, fully cleaved MD39 gp140 (which lacked glycosylation sites at 241 and 289), and a negative control gp120 foldon trimer.

We assessed serum antibody binding in each group to matched antigens at week 26 (two weeks post third vaccination) using enzyme-linked immunosorbent assay (ELISA). All groups demonstrated detectable binding to their corresponding antigens, with the gp120 foldon group exhibiting minimal binding cross-binding to the MD39.2 and MD39.3 antigens (Fig. 1B). We developed base knockout (BaseKO) versions of MD39.2 and MD39.3 that retain the antigenic profiles of the parental antigens while eliminating binding to BG505 base-specific monoclonal antibodies (fig. S2C). Animals immunized with soluble trimers, whether administered as protein or by mRNA-LNPs, exhibited substantially reduced binding to the BaseKO versions of their corresponding antigens compared with the parental forms (Fig. 1B). In contrast, animals immunized with membrane-bound trimers did not demonstrate a significant difference in BaseKO antigen binding (*P* > 0.05; Fig. 1B). By computing the ratio of serum IgG binding to the BaseKO compared to parental forms, we found that a higher proportion of serum antibodies were directed toward the base of the trimer in groups receiving the soluble MD39.2 or MD39.3 gp140 trimers compared with the groups receiving membrane-bound MD39.2 or MD39.3 (*P* = 0.0043 by Mann-Whitney for MD39.2 gp140 vs gp151; *P* = 0.0152 by Mann-Whitney for MD39.3 gp140 vs gp151; Fig. 1C). This indicated that mRNA delivery of membrane-bound trimers substantially refocused responses away from the base.

The impact of filling glycan holes at positions 241 and 289 in BG505 was evident in the autologous neutralization data. Previous studies have demonstrated that rabbits produce high titers of autologous nAbs targeting the 241-glycan hole on BG505 (23, 24). Consistent with expectations, groups immunized with MD39.2 constructs containing glycan holes exhibited higher neutralization titers compared to those receiving MD39.3 constructs, where the glycan holes were filled (Fig. 1D). The BG505 gp120 foldon control immunogen showed minimal or no detectable neutralization.

Focusing only on immunogens with intact glycan holes at 241 and 289, we noted that both gp140 and gp151 variants of MD39.2 delivered by mRNA induced comparable neutralization responses to the MD39 adjuvanted protein group (*P* > 0.05 by Kruskal-Wallis for both MD39.2 gp140 or gp151 mRNA vs MD39 gp140 protein; Fig. 1D). Combined with the finding from ELISA that gp151 elicited reduced base responses compared to gp140 (Fig. 1C), these data suggested that membrane-bound trimers may offer advantages over soluble trimers in the context of mRNA delivery.

### EMPEM revealed base-directed and trimer-degrading responses were elicited by vaccination in rabbits

**Fig. 2.**
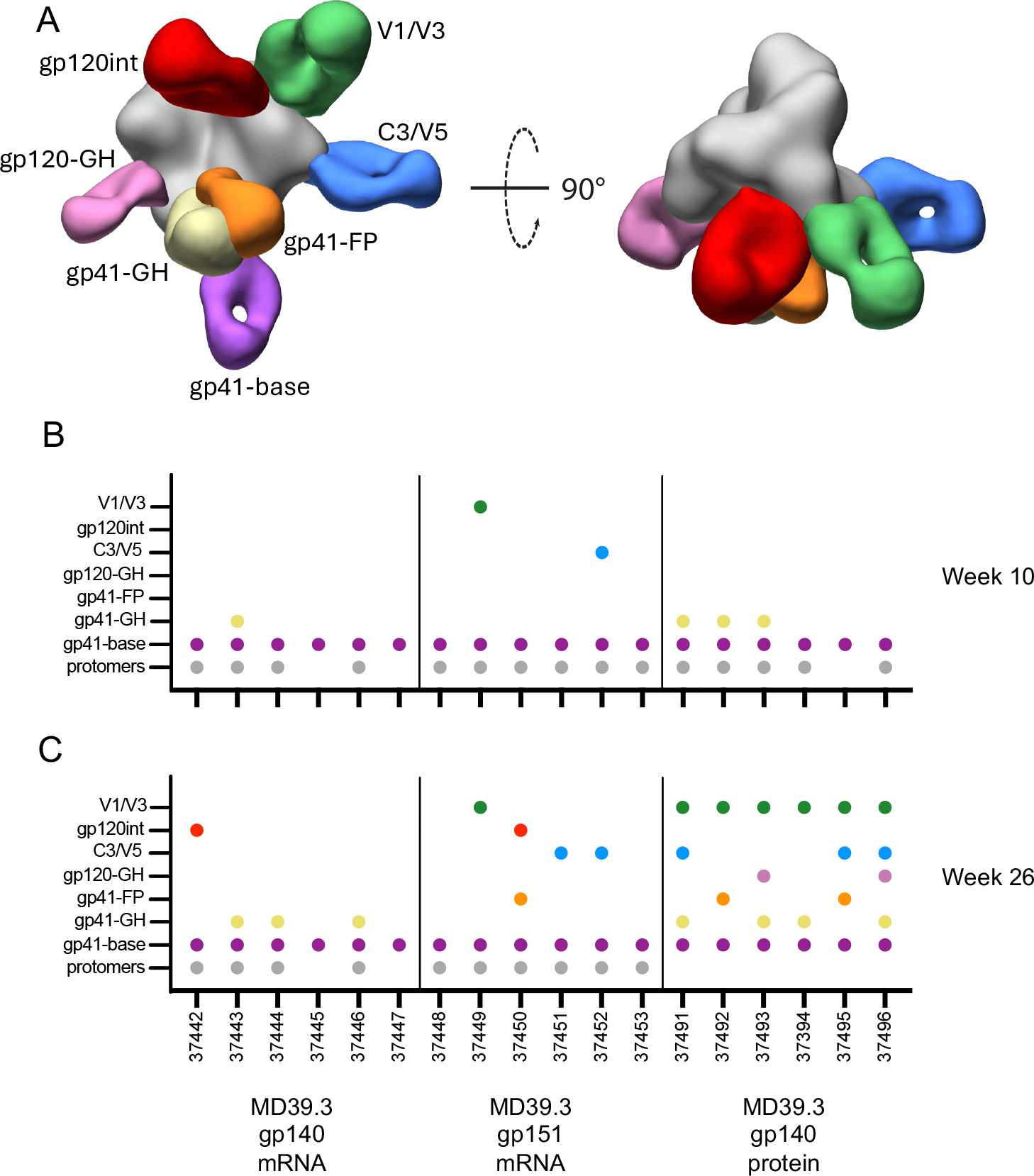
Electron microscopy polyclonal epitope mapping characterized immune responses in rabbit serum. (**A**) Composite 3D map representing the epitopes observed in negative stain EMPEM analysis. (**B and C**) Graphs show the number of animals at week 10 (B) and week 26 (C) with detectable antibodies directed to each specific epitope at 10 and 24 weeks post first immunization. Colored to match (A).

We performed electron microscopy polyclonal epitope mapping (EMPEM) (25) as a method to identify which epitopes on MD39.3 are recognized and targeted by serum antibodies in rabbits immunized with different formulations (Fig. 2A). The immunization groups included MD39.3 gp140 mRNA-LNPs, MD39.3 gp151 mRNA-LNPs, and MD39.3 gp140 protein adjuvanted with SMNP. Analysis of week 10 sera revealed that antibodies from all three groups targeted the base of the trimer (Fig. 2B). Additionally, all groups exhibited antibodies that caused degradation of the MD39.3 gp140 soluble antigen into protomers. The group immunized with the membrane-bound MD39.3 gp151 by mRNA-LNPs demonstrated the most consistent antigen degradation (Fig. 2B). HIV Env trimer-degrading serum antibodies have been previously observed in EMPEM studies (26). In general, some base-binding antibodies are capable of causing trimer degradation and others appear to be non-degrading. Antibody specificities responsible for disassembling the trimer into protomers have previously been shown to bind at or near the Trp clasp (26). When present in substantial amounts, these antibodies pose a challenge for epitope mapping, as the antigen is primarily degraded into protomers. These protomers become saturated with Fabs, complicating their classification through 2D or 3D imaging approaches.

To address the potential complications in the week 26 EMPEM analysis, we used an antigen mixture consisting of 80% MD39.3 gp140 stabilized with internal disulfides (27) and 20% unmodified MD39.3 gp140 (Fig. 2C). The internal disulfides stabilized the trimer by forming interprotomer covalent bonds without altering the antigenicity of the trimer. This antigen mixture enabled the simultaneous detection of antibodies targeting epitopes on the intact HIV Env trimer and those responsible for trimer degradation. The week 26 EMPEM analysis demonstrated that all immunization groups generated base-specific antibodies. Furthermore, the two groups immunized with mRNA-LNP formulations produced trimer-degrading antibodies. In contrast, the group immunized with MD39.3 gp140 protein combined with the SMNP adjuvant did not exhibit trimer-degrading antibodies at week 26 in quantities sufficient to be detected by EMPEM using the antigen mixture strategy (Fig. 2C). To further validate the BaseKO antigens, we performed EMPEM on a subset of animals that received soluble MD39.3, either as a protein or delivered by mRNA-LNPs, using the MD39.3 BaseKO antigen. In all cases, the BaseKO mutations effectively eliminated binding to base-specific serum antibodies (fig. S2D). Moreover, the BaseKO antigen includes an interprotomer disulfide introduced as part of the base knockout mutations, which also conferred the additional benefit of preventing trimer degradation (fig. S2D).

### mRNA-delivered membrane-bound trimers elicited nAbs in NHPs

**Fig. 3.**
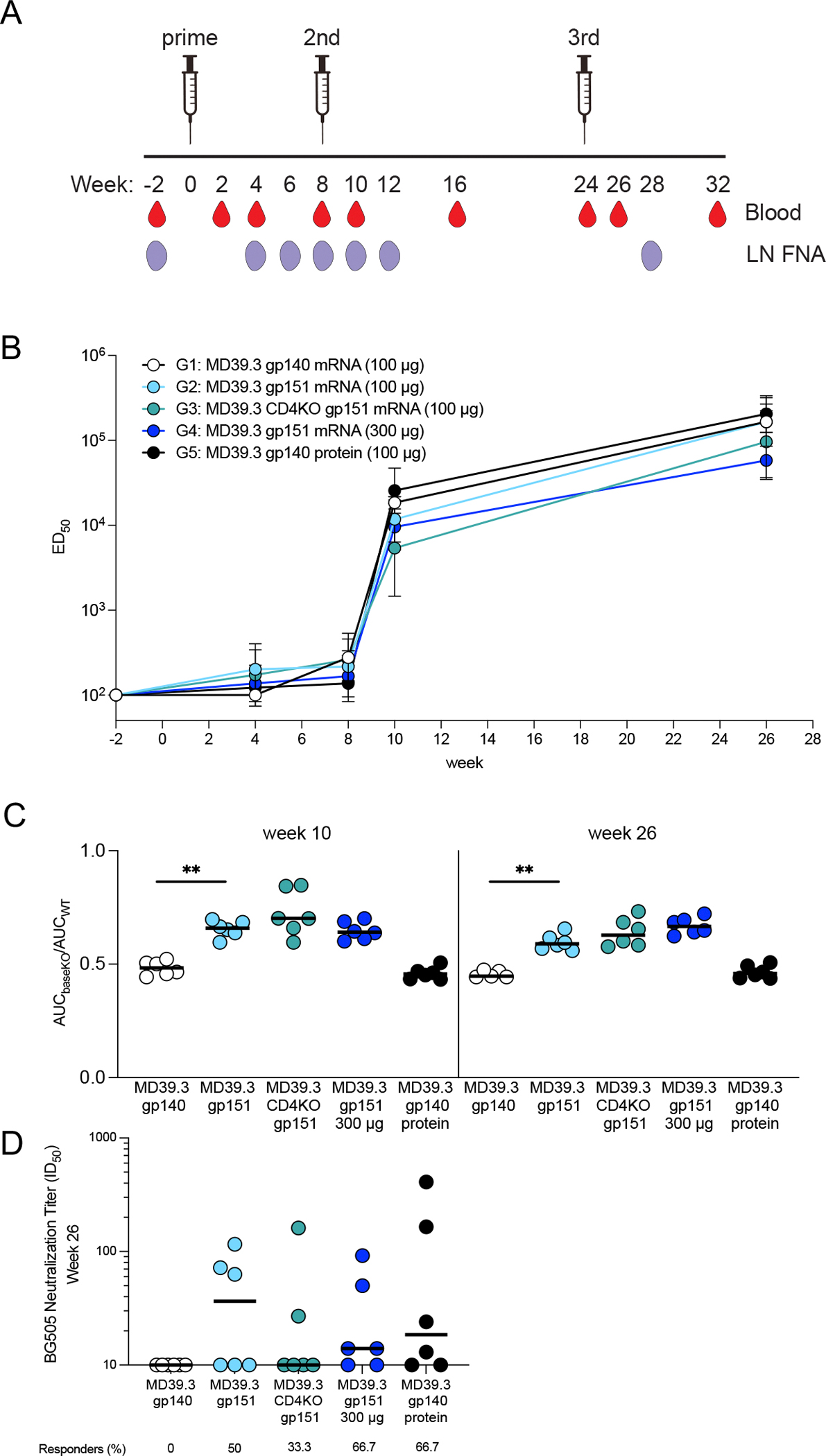
Membrane-bound Env mRNA elicited neutralizing responses in NHPs. (**A**) Five groups of six animals per group were immunized with three doses of BG505 MD39.3 immunogens at weeks 0, 8, and 24. All doses were administered bilaterally with each dose split equally between both sides and delivered intramuscularly into the deltoid muscles. Groups 1 to 4 (G1 to G4) received BG505 MD39.3 mRNA immunogens; Group 5 (G5) received BG505 MD39.3 protein plus SMNP adjuvant. The mRNA groups included: soluble MD39.3 (G1), membrane-bound MD39.3 (G2 & G4) and membrane-bound MD39.3 CD4KO (G3). PBMCs were isolated from whole blood and LN FNA from axillary LNs at the indicated time points. (**B**) Longitudinal ELISA ED_50_ responses are shown for serum antibodies binding to soluble BG505 MD39.3 gp140. Each point is geometric mean ± geometric standard deviation. (**C**) Shown are ratios of AUC values for BG505 MD39.3 gp140 BaseKO antigen over AUC values for BG505 MD39.3 gp140 WT. Lower values indicate the presence of more base binding antibodies in the sera. (**D**) Serum neutralization against BG505 T332N pseudovirus was measured in samples collected at week 26. The frequency of responders is shown below each dataset. Bars indicate median values for AUC measurements and neutralization data. Each point indicates a single animal (n=6 per group) in (C) and (D). Groups 1 and 2 were compared for statistical significance in (C) and (D) using the Mann-Whitney test. Significance levels are indicated as **P < 0.01.

Following demonstration of immunogenicity in rabbits, the MD39.3 immunogens were carried forward to be tested in NHPs. Three immunizations were administered by intramuscular injection bilaterally into the deltoid muscle at weeks 0, 8, and 24 (Fig. 3A). Blood and fine needle aspiration of lymph nodes (LN FNA) were collected throughout the study at different time points. A total of 30 NHPs were immunized, organized into 5 groups with 6 animals per group. Three different immunogen designs were delivered as mRNA-LNP: soluble MD39.3 gp140 (Group 1; 100 εg), membrane-bound MD39.3 gp151 (Group 2 & 4; 100 εg & 300 εg), and membrane-bound MD39.3 CD4KO gp151 (Group 3; 100 εg). Although the HIV envelope trimer does not have measurable affinity for rhesus macaque CD4, the CD4KO construct was included in the study because it was intended for human trials. Group 5 animals received MD39.3 gp140 protein (100 εg) with SMNP adjuvant (750 εg) as a control.

Serum antibodies binding to MD39.3 gp140 were elicited in all groups of NHPs following the first immunization, with concentrations increasing over time and after subsequent immunizations (Fig. 3B). To differentiate non-base responses from base responses, we used the BaseKO probe derived from MD39.3, which was used as a capture antigen alongside regular MD39.3. At weeks 10 and 26, a higher proportion of serum antibodies were directed toward the base of the trimer in groups receiving the soluble trimer (G1 and G5) compared with the groups receiving membrane-bound MD39.3 (*P* = 0.0022 by Mann-Whitney for G1 vs G2 at week 10; *P* = 0.0043 by Mann-Whitney for G1 vs G2 at week 26; Fig. 3C).

No autologous neutralization against the BG505 pseudovirus was observed in the serum samples collected at week 26 from animals immunized with MD39.3 gp140 by mRNA-LNPs. In contrast, all other groups demonstrated animals with detectable autologous neutralization activity at week 26 (Fig. 3D). Eliminating one of the most commonly targeted nAb sites on BG505 Env trimer by filling glycan holes at positions 241 and 289 appeared to substantially reduce the development of nAbs. This was most evident by comparing Group 5 to a historical control group that received 100 εg MD39 gp140 protein (with glycan holes at positions 241 and 289) and 750 εg SMNP by subcutaneous bilateral immunizations above the deltoid (50 εg and 375 εg per limb) at 0, 10, and 24 weeks (22). All animals immunized with glycan hole-containing MD39 protein developed autologous nAbs at week 26, with median titers approximately 19-fold higher than after glycan-hole-filled MD39.3 protein immunization (median 353 vs. 19, *P* = 0.039 by Mann-Whitney; fig. S2E). In sum, membrane-bound MD39.3 mRNA induced detectable neutralizing responses in some animals compared with soluble MD39.3 mRNA, with responding animals achieving titers comparable to those observed following MD39.3 protein immunization aided by a potent adjuvant.

### mRNA-delivered membrane-bound trimer elicited diverse antibody specificities in NHPs

**Fig. 4.**
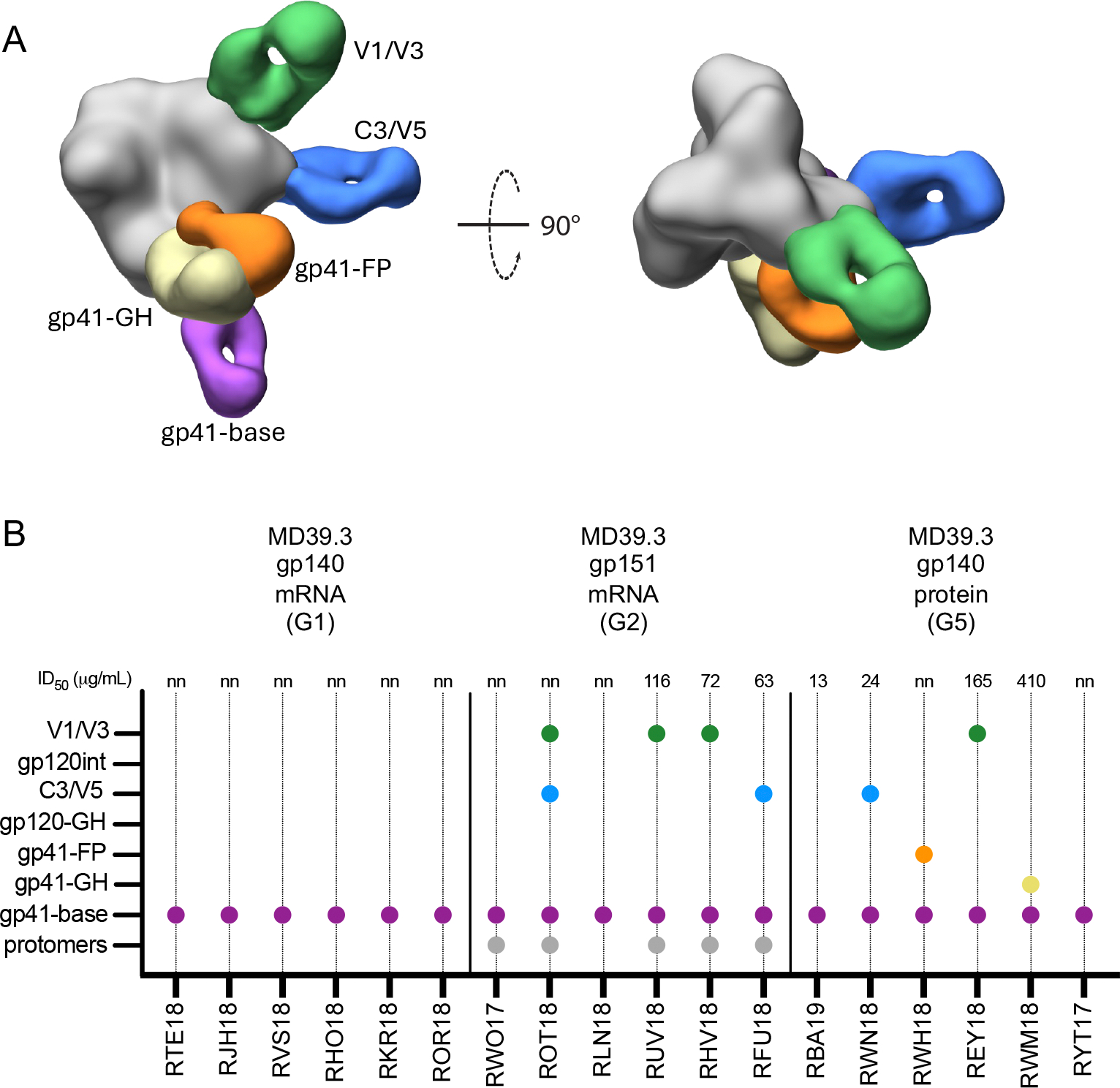
Electron microscopy polyclonal epitope mapping revealed diverse antibody binding modes elicited by membrane-bound MD39.3 mRNA in NHPs. (**A**) Composite 3D map representing the epitopes observed in negative stain EMPEM analysis. (**B**) Graph showing animals with detectable antibodies against each specific epitope at 26 weeks. Circles on the graph indicate antibodies to the epitope were detected. ID_50_ values are listed above the graph; nn, no neutralization. Each animal is indicated on the x-axis. Colored to match (A).

The same antigen mixture strategy used in the rabbit EMPEM analysis was also employed for the week 26 NHP EMPEM analysis testing soluble MD39.3 gp140 mRNA (G1), membrane-bound MD39.3 gp151 mRNA (G2) and MD39.3 gp140 protein (G5). All three groups analyzed by EMPEM demonstrated base-specific antibodies (Fig. 4A). However, only G2, which received membrane-bound MD39.3 gp151 by mRNA-LNPs, exhibited detectable antibodies that caused trimer degradation (Fig. 4B). Additionally, non-base/non-degrading antibodies were identified in both G2 and G5 (Fig. 4B), which likely contributed to the observed autologous neutralization in these groups (Fig. 3D).

### MD39.3 mRNA induced B cell memory responses in NHPs

For evaluation of NHP B cell responses, we first assessed the immune response in axillary LN FNAs to detect total and Env-binding B cell responses (fig. S3A to C). Env-binding germinal center B cells (B_GC_) were detected in some protein immunized animals after 2 to 3 immunizations, but not in the mRNA groups. The total B_GC_ cell frequencies reflected a similar mild to undetectable response typically seen in a non-draining LN, suggesting that the draining LNs were not successfully accessed by the axillary LN FNAs.

**Fig. 5.**
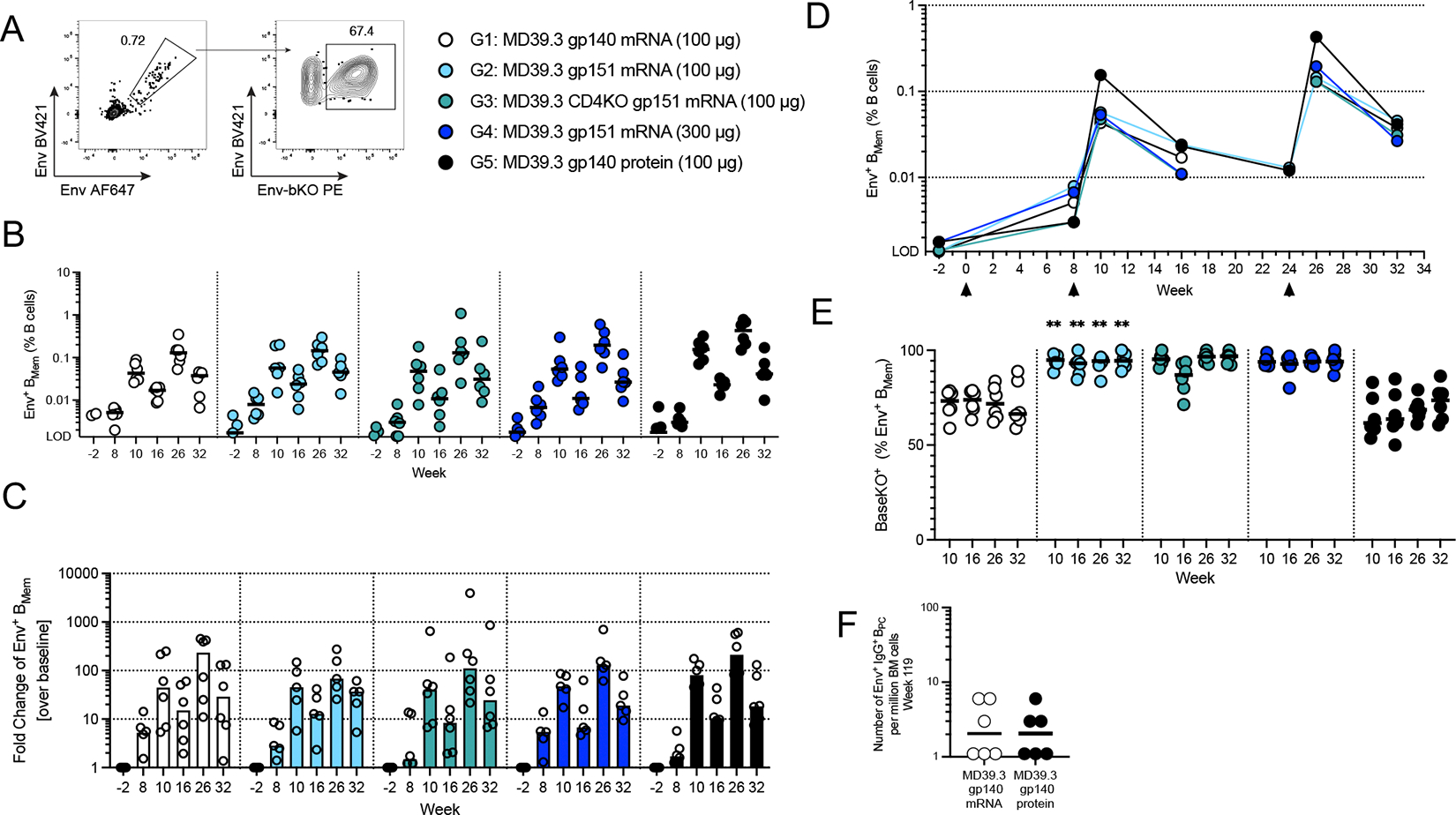
Membrane-bound MD39.3 mRNA induced substantial B cell responses in NHPs. (**A**) Representative flow plots show gating for CD20^+^IgD^−^IgM^−^Env^+/+^ B_Mem_ cells (left) and BaseKO^+^ within Env^+/+^ (right). AF, Alexa Fluor; BV, brilliant violet; PE, phycoerythrin. (**B**) Frequency of Env^+/+^ B_Mem_ cells of total B cells in PBMCs at different time points are shown. Responses that are lower than the limit of detection (LOD, 0.001375) were set at the LOD. (**C**) Fold-change of Env^+/+^ B_Mem_ cells per group and time points over baseline Env^+/+^ B_Mem_ frequencies (pre-immunization week −2) is shown. (**D**) Median frequencies of Env^+/+^ B_Mem_ cells shown in (B) were graphed over time including one additional time point at week 24 (G2 & G5 only). Arrowheads indicate weeks of vaccination. (**E**) Shown are the percentages of BaseKO^+^ Env^+/+^ B_Mem_ cells within total Env^+/+^ B_Mem_ cells in all groups post-boosts. G2 values were compared with corresponding G1 values for statistical significance. (**F**) Shown are the numbers of Env-specific IgG^+^ B_PC_ in bone marrow (BM) aspirates at week 119 (G1 & G5 only). Bars indicate median for B cell frequencies and each point indicates a single animal (n=6 per group) except (D), which shows median of the respective group. Animals with missing baseline samples were excluded from the fold-change analysis in (C). Groups 1 and 2 were compared for statistical significance using the Mann-Whitney test, followed by the Holm-Šídák multiple comparisons test. Significance levels are indicated as **P < 0.01.

We then investigated the memory B cell (B_Mem_) responses in circulation (PBMCs). Env-binding B_Mem_ cells were detectable post-prime and increased with each dose (Fig. 5A and B, fig. S3D). The fold change of Env-binding B_Mem_ cells over baseline surpassed 100-fold for some animals after each boost (Fig. 5C). Env-specific B_Mem_ cells peaked two weeks post-boosts and declined over time (Fig. 5D). Compared with soluble versions of MD39.3, the membrane-bound MD39.3 antigens had higher frequencies of non-base binding B_Mem_ cells (BaseKO^+^) across timepoints ( *P* < 0.01 by Mann-Whitney for G1 vs G2 at weeks 10, 16, 26 and 32 Fig. 5E). Bone marrow aspirates were collected from select available groups (G1 & G5) more than 1 year since the last immunization. Env-specific bone marrow plasma cells (B_PC_) were detectable for 3 animals per group for G1 and G5 (Fig. 5F). In summary, membrane-bound MD39.3 mRNA vaccines induced Env-binding B_Mem_ responses after 2 and 3 doses, with lower frequencies of Env base-binding B_Mem_ cells compared with soluble Env trimer protein or mRNA immunizations.

### MD39.3 mRNA vaccination induced antigen-specific T cell responses in NHPs

**Fig. 6.**
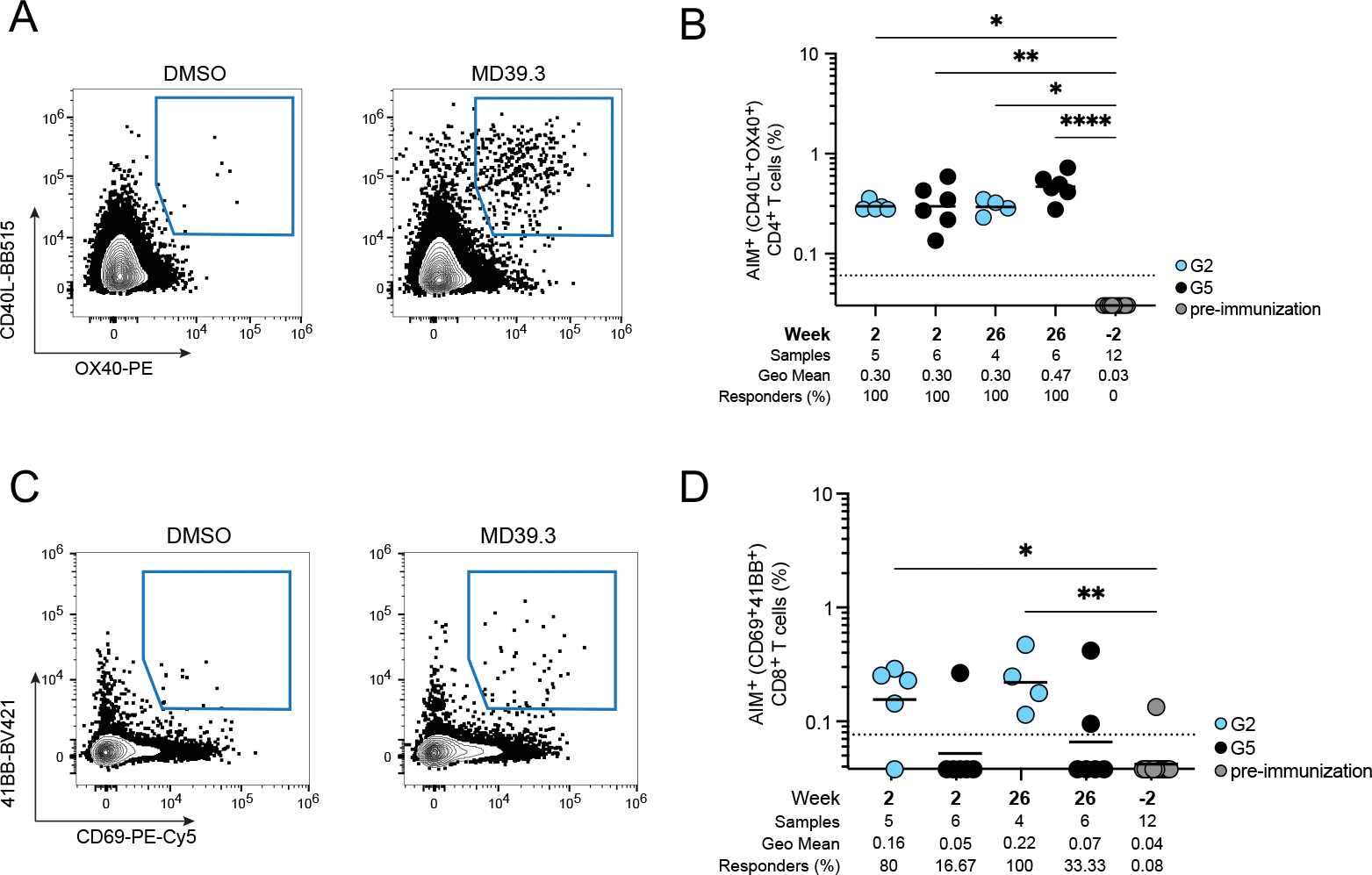
Membrane-bound MD39.3 mRNA induced both CD4^+^ and CD8^+^ T cell responses in NHPs. (**A**) Representative flow cytometry plots show gating for Env-specific AIM^+^ CD4^+^ T cell responses after stimulation with control DMSO or with MD39.3 peptides; BB, brilliant blue. (**B**) Env-specific AIM^+^ CD4^+^ T cell responses are shown for G2 and G5 at week 2 (post-prime), week 26 (post-third dose) and week −2 (pre-immunization). (**C**) Representative flow cytometry plots show gating for Env-specific AIM^+^ CD8^+^ T cell responses after stimulation with control DMSO or with MD39.3 peptides; Cy, cyanine. (**D**) Env-specific AIM^+^ CD8^+^ T cell responses are shown for G2 and G5 at week 2 (post-prime), week 26 (post-third dose) and week −2 (pre-immunization). Data are shown as background subtracted. Non-responder samples were set at baseline. The dotted black line indicates the limit of quantification (LOQ). Bars represent geometric mean and each point indicate a single animal (n=6 per group, except for G2 which had n = 5 at week 2 or n = 4 at week 26). Pre-immunization had animals from both G2 and G5 (n = 12). The number of samples, the geometric mean (Geo Mean) and the frequency of responders are shown below the plots in (B) and (D). Animals with missing samples or samples exhibiting poor viability were excluded from the analysis. Statistical significance was assessed using the Kruskal-Wallis test, followed by Dunn’s multiple comparisons test. Significance levels are indicated as *P < 0.05, **P < 0.01, and ****P < 0.0001.

Based on the B cell and neutralizing responses, we focused our remaining analysis on a single mRNA group: the membrane-bound MD39.3 gp151 mRNA at the 100 εg dose, compared with the adjuvanted MD39.3 protein group. This choice was driven by the superior non-base responses of MD39.3 gp151 compared with the soluble gp140 form, with no obvious benefit at higher doses (300 εg) or from the CD4KO mutation in rhesus macaques. Thus, we tested the capacity of membrane-bound MD39.3 gp151 mRNA (Group 2) and MD39.3 gp140 protein (Group 5) to induce T cell responses post-prime (week 2) and post-boost (week 26) by activation-induced marker (AIM) and intracellular cytokine staining (ICS) assays. Both groups induced similarly robust Env-specific AIM CD4^+^ T cell responses in circulation (*P* = 0.8918 by Mann-Whitney for G2 vs G5 at week 2; *P* = 0.0667 by Mann-Whitney for G2 vs G5 at week 26; Fig. 6A and B, fig. S4A). The combination of AIM plus ICS showed that priming with mRNA-delivered membrane-bound MD39.3 generated Env-specific CD4^+^ T cells capable of producing interferon (IFN)-γ, tumor necrosis factor (TNF), granzyme B, or interleukin (IL)-2 (fig. S4B). Env-specific circulating T follicular helper (cT_FH_) cells were induced by mRNA-delivered membrane-bound MD39.3 priming to frequencies comparable to protein immunization (fig. S4C). Env-specific CD8^+^ T cell responses were detected in the majority of the mRNA-delivered membrane-bound MD39.3 animals (80% to 100% of animals in G2 were responders, compared with 16.67% to 33.33% in G5; *P* = 0.0996 by Mann–Whitney test for G2 vs. G5 at week 2; *P* = 0.0524 by Mann–Whitney test for G2 vs. G5 at week 26; Fig. 6C and D). Overall, after a single immunization, mRNA-delivered membrane-bound Env generated detectable CD8^+^ T cell responses and consistent Env-specific CD4^+^ T cell responses with diverse functionalities.

### Sequencing of antigen-binding B_Mem_ cells from vaccinated NHPs revealed mutational and clonal dynamics

**Fig. 7.**
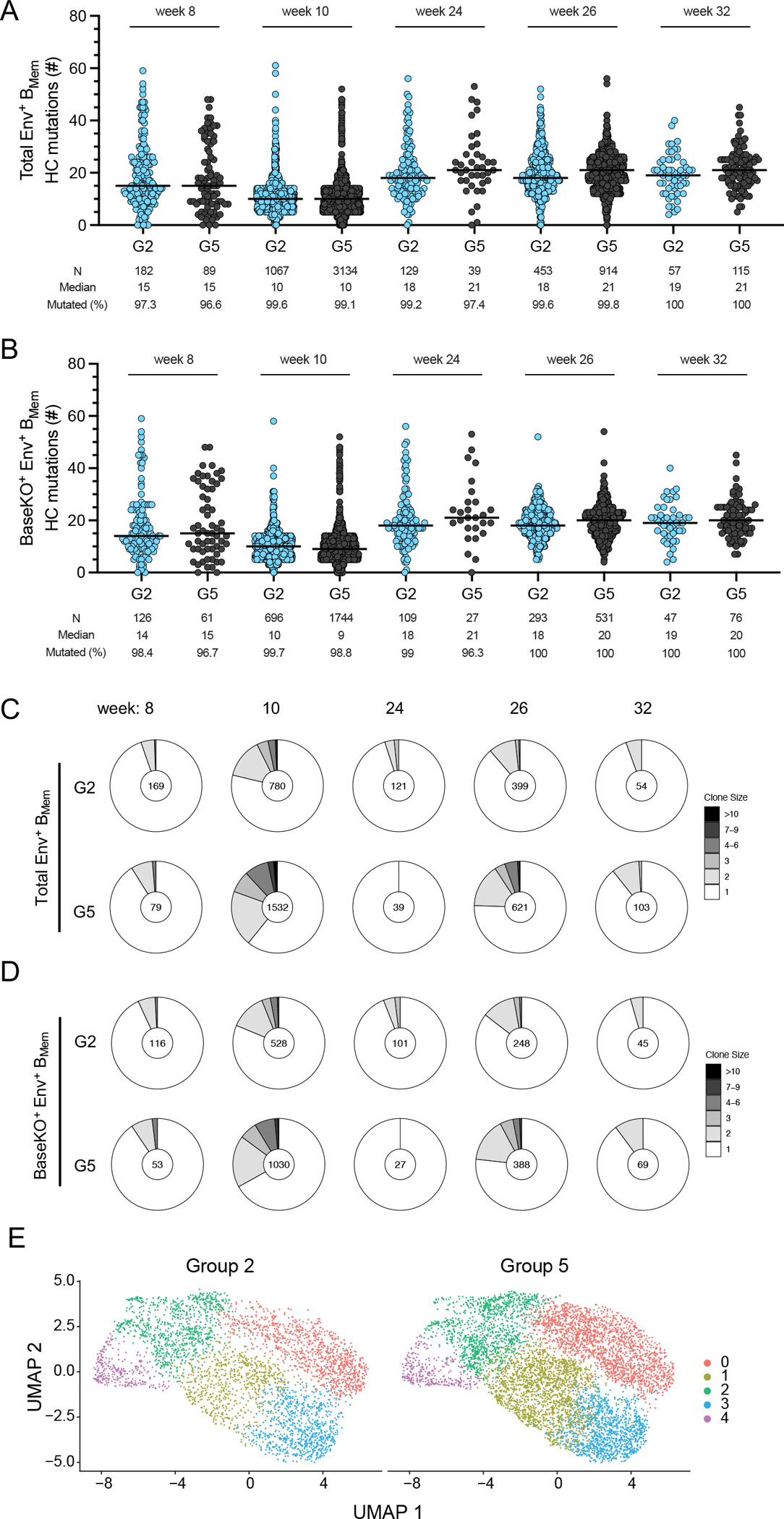
Single cell analysis of MD39.3-binding B_Mem_ cells indicated somatic hypermutation in NHPs. (**A**) Heavy chain mutations in total Env-binding B_Mem_ cells were assessed for G2 and G5 at week 8, 10, 24, 26 and 32. (**B**) Heavy chain mutations on non-base-Env-binding B_Mem_ cells (BaseKO^+^) were assessed for the same groups and timepoints as in (A). Bars indicate median for mutational analysis and each point indicates a single B cell sequence. The number of B cell sequences analyzed, the median number of HC mutations, and the frequency of mutated HCs are shown below each plot. (**C**) Total Env-binding B_Mem_ clonal families were analyzed for groups 2 and 5 across timepoints. Numbers within the Donut plot indicate total number of clonal families detected for each measurement. (**D**) Non-base-Env-binding B_Mem_ clonal families were analyzed for the groups and timepoints as shown in (C). **(E**) Uniform manifold approximation and projection (UMAP) visualization of single-cell gene expression profiles identifying clusters among Env-binding B_Mem_ cells sorted from PBMCs for G2 and G5.

Single B cell receptor (BCR) sequencing was utilized to investigate the sequences and mutational patterns of Env-binding B_Mem_ clones. We sorted and sequenced Env-binding B_Mem_ cells in PBMCs from mRNA-delivered membrane-bound MD39.3 immunized animals (Group 2) and MD39.3 protein immunized animals (Group 5) at five timepoints. At week 8 post-prime, Env-binding B_Mem_ cells exhibited relatively high rates of SHM in both vaccination groups, with over 95% of the recovered Env-binding B_Mem_ cells being mutated (Fig. 7A). Two weeks post-boost, (week 10) the frequencies of Env-binding B_Mem_ cells increased substantially (Fig. 5B and D, Fig. 7A), with more than 99% of cells exhibiting SHM. Median SHM in Env-binding B_Mem_ cells was greater at week 24 than week 10 for animals in both vaccination groups (*P* < 0.0001 by Mann-Whitney for both groups 2 and 5; Fig. 7A). Two weeks after the third dose (week 26), SHM of the Env-binding B_Mem_ cells was similar to week 24. The robust induction Env-binding B_Mem_ cells with SHM indicates that germinal centers were induced in these animals, but the draining LN was not sampled in the FNAs. Overall, Env-binding B_Mem_ cells accumulated substantial SHM in their BCRs after the prime and boost immunizations, indicating ongoing affinity maturation.

We expressed several monoclonal antibodies (mAbs) from select BCR sequences of Group 2 animals at week 26 and mapped their epitope specificities using surface plasmon resonance (SPR) and a panel of mutant MD39.3 antigens (fig. S5A). Among the mAbs that bound to MD39.3, three targeted the base region, two targeted the C3/V5 region, and two targeted the V1/V3 region. Other mAbs exhibited high-affinity binding to MD39.3 but could not be mapped to a specific epitope or region. mAbs with an affinity of 100 nM or better for MD39.3 were further assessed for neutralization potential. Only the two mAbs targeting the V1/V3 region demonstrated the ability to neutralize autologous BG505 pseudovirus and several epitope mutants (fig. S5B). The lack of neutralization despite high-affinity binding to MD39.3 observed for many mAbs suggested that these mAbs may be recognizing peptide epitopes present on MD39.3 but absent or shielded on the BG505 pseudovirus (such as Link14 or the V3 tip). To address this potential, we assessed the ability of the mAbs to bind to a V3 peptide or the Link14 peptide and found that none of the mAbs bound either peptide (fig. S5C). Overall, the immunogen induced mAbs with affinities in the nanomolar range, including some with neutralizing capabilities.

Inclusion of a barcoded BaseKO probe in the B_Mem_ sorts allowed discrimination between Env base-binder and non-base binder B_Mem_ cells. Non-base-binding or base-binding B_Mem_ cells possessed similar SHM kinetics compared to total Env-binding B_Mem_ cells, with > 95% of non-base-binding B_Mem_ cells mutated post-prime (Fig. 7B, fig. S6A). The interclonal diversity of Env-binding B_Mem_ cells was high. Larger clonal families were detected at the peak post-boost timepoints at week 10 and 26 (Fig. 7C and D). V gene usage was overall similar between both groups (fig. S6B), as were heavy chain complementarity determining region 3 (CDRH3) lengths (fig. S6C). In addition, phenotypic characteristics of sorted Env-specific B_Mem_ were assessed by whole transcriptomic gene expression profiling, comparing mRNA versus protein immunization. Five B_Mem_ clusters were identified (fig. S7). All five B_Mem_ clusters were represented after mRNA or protein immunization (fig. 7E), as well as at every time point (fig. S7). In sum, non-base-binding B_Mem_ cells accumulated substantial SHM in their BCRs after prime and boost immunizations.

## Discussion

No HIV vaccine has been approved to date, and major efforts are focused on inducing protective immune responses through bnAbs. However, naïve (germline) precursor B cells capable of maturing into bnAbs are typically rare and rely on competitive recruitment into the germinal center response. Germline-targeting immunogens represent a promising approach for the initial recruitment of these rare B cell precursors (4, 18, 28, 29). Following successful recruitment, the newly differentiated B_GC_ and B_Mem_ cells require sequential immunizations with increasingly native-like immunogens to guide them through rounds of clonal expansion and SHM. This process aims to ultimately refine the bnAb precursors into functional bnAbs. The membrane-bound immunogen MD39.3 tested here exhibits features such as base occlusion and redirection of antibody responses, which may be beneficial for immunogen design and could support its potential use at later stages of complex HIV vaccine strategies, including germline-targeting approaches.

Immunization with membrane-bound trimers elicited base-specific serum antibodies, as detected by EMPEM and ELISA, though the magnitude of this response was lower than that induced by soluble trimers. The base-specific antibodies generated by membrane-bound trimers were predominantly trimer-degrading, targeting sites near the Trp clasp. Previously published trimer degrading antibodies exhibited variable angles of approach, binding to the trimer both parallel (30, 31) and perpendicular (26) to the membrane. Immunization with membrane-bound trimers may have elicited trimer-degrading, base-specific antibodies that approach the trimer parallel to the membrane. The relevance of the induction of trimer-degrading antibodies detected by EMPEM to the induction of nAbs in vivo remains unclear.

The data suggest that MD39.3 mRNA can act as an effective immunogen in preclinical mammalian models, with membrane-bound expression likely favoring subdominant epitope responses over the soluble form. Neutralizing responses in both rabbits and NHPs were low to undetectable following MD39.3 gp140 immunization, in contrast with the measurable responses elicited by the gp151 membrane-bound version. Similarly, EMPEM did not reveal V5/C3 or V1/V3 targeting following gp140 immunization, whereas these autologous nAb-associated sites were targeted in NHPs after gp151 immunization. The comparison between mRNA and protein underscores the high standard set by decades of research dedicated to optimizing protein vaccine design and adjuvant strategies. In this study, immunization with membrane-bound MD39.3 mRNA elicited B_Mem_, nAb, and CD4^+^ T cell responses comparable to those achieved with MD39.3 protein formulated with SMNP adjuvant. Additionally, no concerning events were observed in any of the animals. Although mRNA-LNP platforms continue to be optimized in terms of targeted cellular delivery, control over antigen expression kinetics, and the intrinsic immunostimulatory properties of the LNP, the current membrane-bound MD39.3 mRNA construct offers several advantages over MD39.3 protein, including: (1) eliminating the need for co-formulated adjuvants, (2) reducing exposure of the trimer base, and (3) simpler and more cost-effective manufacturing. Our findings demonstrate that membrane-bound MD39.3 mRNA represents a promising platform for vaccine development strategies and suggest more generally that for mRNA delivery of HIV trimers at any stage of a germline-targeting vaccine regimen, membrane-bound trimer is likely preferred over secreted soluble trimer.

A central value of NHPs as a vaccine development model is their evolutionary and immunological relatedness to humans. However, direct comparable studies of immune responses in NHPs and humans are rarely available. HVTN302 is a clinical trial comparing the same MD39.3 mRNA immunogens in humans, with comparable doses and immunization regimens (16, 17). The immune responses in NHPs after mRNA MD39.3 immunizations were consistent with those subsequently found in humans. Strong B_Mem_ responses were induced in both NHPs and humans after two immunizations. In both NHPs and humans immunized with membrane bound MD39.3, a majority of the B_Mem_ were specific to non-base epitopes. The B_Mem_ acquired SHM over time in both NHPs and humans. Strong CD4^+^ T cell responses were generated in almost all cases, in both NHPs and humans, and some CD8^+^ T cell responses were detected in MD39.3 mRNA immunized NHPs and humans. Most notably, autologous nAbs were generated in both NHPs and humans, with similar frequencies of responders after three immunizations between the species for 100 εg soluble MD39.3 and 100 εg membrane-bound MD39.3 gp151. Humans appeared to generally make higher BG505 autologous nAb titers. Moreover, a majority of the human BG505 nAb response targeted a V1 epitope, which was not observed in the NHPs, suggesting that NHPs are a somewhat more difficult model for BG505 nAb development.

A comparison here to historical NHP MD39 immunization data indicated that the glycan hole filling of MD39.3 makes a substantially more challenging target for BG505 autologous nAb generation, which is a useful reference point when comparing across diverse BG505 Env trimer immunization study results, including in humans. Additionally, MD39.3 protein formulated with SMNP as an adjuvant was able to induce subdominant responses despite a high frequency of base-binding responses. This is likely due to the potency of SMNP, which has been shown to enhance immune responses by increasing lymphatic drainage and antigen retention in draining LNs, thereby promoting stronger germinal center responses and improved neutralization (22, 32).

The robust detection of Env-binding B_GC_ cells in some animals exclusively in the protein immunization group (Group 5) may indicate variability in NHPs in LN drainage and localized immune responses between vaccine platforms. Previous studies have suggested that intramuscularly injected immunogens exhibit more restrictive LN drainage compared with subcutaneous administration (33, 34). Nonetheless, the data herein indirectly demonstrate robust germinal center responses occurred after mRNA priming and boosting, as evidenced by almost all Env-binding B_Mem_ exhibiting SHM, with SHM rates comparable to those for protein immunization plus SMNP, an adjuvant that has been shown to induce robust germinal center responses (4, 22, 35, 36). Previous studies have shown that intramuscularly-delivered COVID-19 mRNA vaccines induce robust and long-lasting germinal center responses in humans (37). However, anatomical differences in LN drainage between humans and NHPs may exist. Buckley *et al*. recently demonstrated that LN drainage following intramuscular mRNA vaccination in NHPs may differ from that of protein immunization and instead involves deeper LNs (38) that are inaccessible by FNAs. Long-lasting germinal center responses to a priming immunization are preferred for enhancing affinity maturation and promoting competitive engagement of rare B cell clones targeting subdominant epitopes (35, 39, 40). Ongoing investigations aim to better understand how mRNA vaccines drain in NHPs and inform on germinal center responses following immunization.

We observed a strong induction of Env-binding B_Mem_ cells following immunization. Peak responses were detected in NHPs two weeks after each boost, followed by a decline in subsequent weeks, consistent with antigen-binding B_Mem_ kinetics in NHPs after two doses of a COVID-19 mRNA vaccine (41). This suggests that NHPs may exhibit distinct B_Mem_ kinetics compared with humans, possibly due to a pronounced but short-lived expansion of B_Mem_ cells shortly after boosting, regardless of the vaccine platform.

Both membrane-bound MD39.3 mRNA and protein immunizations induced Env-specific CD4^+^ T cell responses. A greater number of animals mounted Env-specific CD8^+^ T cell responses in the membrane-bound mRNA group comparable to the protein group. This is consistent with the observation that mRNA delivery enhances CD8^+^ T cell priming, as seen with COVID-19 vaccines (42–44), and with MD39.3 mRNA in humans (16), potentially due to antigen synthesis within professional antigen-presenting cells (45). The presence of Env-specific CD8^+^ T cells in mucosal tissues could synergize with nAbs to provide protection against HIV infection (46). These responses may also be useful in therapeutic vaccines. Thus, understanding how to induce and enhance antigen-specific CD8^+^ T cell responses elicited by mRNA vaccines remains an important goal for future research.

In addition to limitations discussed above, including the ongoing optimization of mRNA-LNP platform and the undetectability of draining LN responses by FNAs, EMPEM provided qualitative insights into various antibody-antigen binding modes but did not provide quantitative data on the proportion of the overall immune response targeting a specific site. We observed antibody responses capable of dissociating the Env trimer into protomers in both rabbits and NHPs. In rabbits, these responses were induced by soluble mRNA, membrane-bound mRNA, and protein; however, these “trimer degrading” responses were most pronounced in the membrane-bound mRNA group. In NHPs, trimer-degrading antibody responses were detected exclusively in the membrane-bound mRNA group, but not in the soluble mRNA or protein groups. Collectively, our findings, along with previous reports, suggest that these antibody responses can be induced across different vaccine platforms. However, membrane-bound antigen expression appears to present the antigen in a conformation that better exposes epitopes capable of eliciting these specific antibodies. Ongoing investigations are examining whether these antibody responses negatively affect the integrity of membrane-bound Env trimers. Additionally, as a native-like immunogen, MD39.3 alone is insufficient to elicit broad neutralization and will likely need to be incorporated into more complex immunization strategies that at least include germline-targeting priming immunogens.

In sum, these preclinical study results support the continued development of membrane-bound MD39.3 and other Env trimers delivered by mRNA. The human use of the mRNA-encoded MD39.3 has been clinically evaluated in HVTN302 (16, 17).

## Materials and Methods

### Study design

The primary objectives of this study were to investigate the preclinical immunogenicity of BG505 MD39.3 HIV envelope trimer delivery using mRNA-LNP in mammalian models. Rabbits were used as a small mammalian animal model, whereas NHPs served as a physiologically relevant model for humans to assess translational potential. The MD39.3 mRNA immunogens were tested in two formats: soluble and membrane-bound. All animal procedures complied with NIH guidelines and were approved by the respective Institutional Animal Care and Use Committees (IACUCs). Group sizes were predetermined to include six animals per group, based on prior power analysis (47), to ensure sufficient statistical power for reliable measurement of immunological responses and detection of meaningful differences in a single experiment. The population from which the animals were selected was defined based on availability and suitable age groups for the study. Animals were randomly assigned to experimental groups while ensuring balanced sex representation. The study was not blinded. Data collection continued until all planned endpoints were reached. The animals were monitored daily by clinical veterinarians, and physical examinations were conducted during each anesthetic access. No concerning events were reported in the animals. All animals, including outliers, were included in the analysis, with the exception of animals with poor viability PBMCs in the T cell analysis (Fig. 6 and fig. S4). Primary endpoints included safety and immunogenicity measures such as antibody titers, neutralization responses, and antigen-specific B and T cell responses.

### Protein production

BG505 MD39.2 gp140 and BG505 MD39.3 gp140 trimer immunogens; His-tagged trimer ELISA, SPR, and EMPEM reagents; and His-Avi-tagged biotinylated trimer probes were produced by transient co-transfection of HEK-293F cells (Thermo Fisher) as previously described (21). For BG505 MD39 gp140, co-transfection using a plasmid encoding human Furin was performed to ensure gp120/gp41 cleavage as previously described (21). His-tagged and His-Avi-tagged trimers were purified by immobilized metal ion affinity chromatography (IMAC) using HisTrap excel columns (Cytiva) followed by size-exclusion chromatography (SEC) using a Superdex 200 Increase 10/300 GL column (Cytiva). His-Avi-tagged trimers were biotinylated using BirA (Avidity) according to the manufacturer’s instructions and purified again to remove excess biotin using SEC. BG505 MD39 gp140, BG505 MD39.2 gp140, and BG505 MD39.3 gp140 trimers were purified by 2G12 affinity chromatography followed by SEC. Immunogen preps were confirmed to contain < 5 EU/mg of endotoxin using an Endosafe instrument (Charles River). Monoclonal IgG antibodies were produced in house as previously described (48). Monoclonal Fabs were produced and purified as previously described (4). The following mutant versions of BG505 MD39.3 were produced for use in ELISA, BLI, EMPEM, SPR, and flow cytometry: (1) BG505 MD39.3 CC5 (BG505 MD39.3 + E49C-L555C and A73C-P561C); (2) BG505-MD39.3 V1/V3-KO (BG505 MD39.3 +133aN and 136aA); (3) BG505-MD39.3 C3/V5-KO (BG505 MD39.3 + T461N, S463T, T464N, and E466S); (4) BG505-MD39.3 BaseKO sorting probe (BG505 MD39.3 + R500A, Q658K, and A662E); and (5): BG505-MD39.3 BaseKO SPR, ELISA, and EMPEM probe (BG505 MD39.3 + R500A, C605T, Q658T, L660N, A662T, L663C, D664N, 665G, and 666T).

A BaseKO version of BG505-MD39.2 was also produced for use in ELISA and BLI.

### Cell surface antigenicity

Cell surface antigenicity of membrane-bound HIV Env trimers was assessed using flow cytometry. 25 μg of plasmid DNA encoding membrane-bound HIV Env trimers in 833 μl of Opti-MEM media (Thermo Fisher) was added to 833 μl of Opti-MEM media (Thermo Fisher) containing 50 μl of 293Fectin (Thermo Fisher), mixed and incubated at room temperature for 15 to 20 minutes. Transfection reactions were added to 25 mL of HEK293F cells at 1 million cells/mL in FreeStyle 293F media. All transfections were carried out in duplicate. Transfected cells were incubated at 37°C and 8% CO_2_ on an orbital shaker platform at 125 rpm for 2 days. Transfected cells were transferred to deep-well 96-well plates (1mL per well) and spun down at 650 x g for 5 minutes. Cells were resuspended in 110 μl FACS buffer, consisting of 2% fetal bovine serum (FBS) in phosphate buffered saline (PBS), containing 10 μg/mL primary Fabs (PGT145, PGT151, VRC01, PGT121, PGT128, 10E8, F105, B6, 19b, or PBS control), transferred to U-bottom 96-well plates, and incubated on a plate shaker for 1 hour at 4°C. Cells were washed twice with FACS buffer and resuspend in 50 μl FACS buffer containing 0.05 μl SYTOX Green Dead Cell Stain (Thermo Fisher) and 0.25 μl AF647 AffiniPure Goat Anti-Human IgG F(ab’)₂ fragment specific (Jackson ImmunoResearch, Catalog #109–605-006). Stained cells were incubated for 20 minutes at room temperature in the dark and washed twice with FACS buffer. Cells were resuspended in 150 μl FACS buffer. Cells were analyzed on a NovoCyte 3000 flow cytometer (Agilent) using BD FACS Diva 6 software (BD Biosciences). Approximately 50,000 live cells were acquired per well. Data were analyzed using FlowJo v10.8 and plotted using Prism (v10.4.1; GraphPad).

### BLI

Soluble gp140 immunogen antigenicity was assessed using BLI as previously described (49) using a panel of bnAbs and non-neutralizing mAbs. A soluble BG505 gp120 foldon trimer was used as a control to show the antigenic profile of a non-native trimer (low binding to quaternary specific bnAbs and high binding to non-neutralizing mAbs). BaseKO versions of MD39.2 and MD39.3 were also assessed for antigenicity using BLI and including the base-specific mAbs RM19R, RM20A3, and RM20B1 (50). The impact of binding human CD4 on the antigenicity of the soluble gp140 immunogens was assessed using BLI. Biotinylated versions of BG505 SOSIP, BG505 MD39, BG505 MD39.3, and BG505 MD39.3-CD4KO were captured on streptavidin biosensors (Satorius) at 25 εg/mL in kinetics buffer containing 1X PBS, 0.01% (w/v) bovine serum albumin (BSA), and 0.002% (v/v) Tween-20 until a response shift of 0.5 nm was reached. After acquiring a baseline in kinetics buffer alone, biosensors were transferred to wells containing 2 εM human soluble CD4 (sCD4) (51) or kinetics buffer alone and allowed to saturate for 2 minutes. The biosensors were transferred to wells containing 2 εM 17b IgG or kinetics buffer alone for additional 2 minutes to assess binding of 17b IgG after CD4 induced conformation change. For the peptide BLI experiment, biotinylated versions of the Link14 (VGSHSGSGGSGSGGHAAAGGAGK-biotin) and V3 (TRPNNNTVKSIRIGPGQAFYYTGGAGK-biotin) peptides were produced by GenScript and loaded onto streptavidin biosensors (Satorius) at 10 εg/mL in kinetics buffer. After acquiring a baseline in kinetics buffer alone, biosensors were transferred to wells containing 1 εM of various IgG antibodies in kinetics buffer. The V3 specific mAb 19b was included as a positive control along with negative control bnAbs BG18 and PGT145.

### nsEM

MD39.3 SOSIP was diluted in Tris-buffered saline (TBS) to 0.02 mg/mL and adsorbed onto a carbon-coated and plasma cleaned copper mesh electron microscopy (EM) grid. Following staining for 45 seconds with 2% (w/v) uranyl formate, approximately 100 micrographs were collected on a Thermo Fisher Talos F200C equipped with a Ceta 16M camera using EPU software. Particles were picked, extracted, and subjected to 2D classification using Relion 4.0 (52). The number of native-like trimers was calculated by summing particles belonging to 2D class with clear, compact trimers and dividing by the total number of particles in the 2D classification job. For BG505 or MD39.3 complexes with human sCD4, each trimer was incubated with 6X molar excess of sCD4 to trimer for 3 hours at room temperature. Grid preparation, data collection, and analysis steps were identical to those above for MD39.3 alone.

### Glycan Occupancy

Site specific glycan profiling for BG505 MD39.3 gp140 was conducted as previously described (53). Site specific glycan profiling data for BG505 MD39 gp140 was adapted from (53) with permission. The degree of glycan occupancy and proportion of glycans that were complex and oligomannose/hybrid type were determined.

### mRNA immunogens

Amino acid sequences encoding BG505 MD39.2 and BG505 MD39.3 immunogens (gp140 and gp151) were provided to Moderna for production as mRNA-LNP immunogens. Immunogens were supplied as mRNA-LNPs at an mRNA concentration of 0.5 mg/mL. LNPs included 4 lipid excipients: the proprietary ionizable amino lipid SM-102 and three commercially available lipids: cholesterol, DSPC, and PEG2000 DMG. mRNA-LNP vaccines were formulated in 20 mM Trometamol (Tris) buffer, 87 g/L sucrose and 10.7 mM sodium acetate at a pH of 7.5. mRNA-LNP vaccines were sterile filtered and filled into RNAse-free glass vials at a mRNA concentration of 0.5 mg/mL.

### Animals and immunizations

Female New Zealand white rabbits (ages at start of study: range, 2.8 to 33 months; average age, 6.0 months; weights at start of study: range, 1.6 to 4.1 kg; average weight, 2.7 kg) were housed and immunized at Pocono Rabbit Farm. Animals were immunized intramuscularly (IM) with a single 500 εl injection in the right thigh. Doses per injection were: 30 εg (protein), 375 εg (SMNP adjuvant), 100 εg (mRNA-LNP), all diluted in PBS. This animal experiment was conducted in compliance with the Animal Welfare Act Regulations (9 CFR), U.S. Public Health Service Office of Laboratory Animal Welfare (OLAW) Policy on Humane Care and Use of Laboratory Animals, and AAALAC accreditation. The immunization protocol (20G131) was reviewed and approved by the Animal Care and Use Committee of the Pocono Rabbit Farm and Laboratory.

NHP study animals were 3 to 4 years old male and female with a weight range of 5.2 to 7.4 kg Indian-origin rhesus macaques (*Macaca mulatta*). Animals were bred and maintained at Emory National Primate Center at Emory University in strict accordance with protocols 202000087 and 2021000197 approved by the Animal Care and Use Committee (IACUC) at Emory. Animal care facilities are accredited by the U.S. Department of Agriculture (USDA) and the Association for Assessment and Accreditation of Laboratory Animal Care (AALAC) International. Animals were tested negative for simian immunodeficiency virus. All immunizations were administered IM into the left and right deltoid muscle. The total dose for all MD39.3 mRNA vaccines was 100 εg (except for Group 4, where the dose was 300 εg). The total dose for MD39.3 protein was 100 εg plus 750 εg SMNP adjuvant. SMNP was produced as previously described (22). Blood and LN FNAs were collected at indicated time points from the animals. PBMCs and plasma were separated from whole blood and serum was collected independently. Bone marrow aspirates were performed only on animals from the soluble mRNA group (G1) and the protein group (G5), as these animals were returned to the primate colony at the end of the study and subsequently enrolled in an unrelated study. Although animals from other groups were not available for this procedure, data from G1 and G5 were included due to their relevance and value. Animals were treated with anesthesia (ketamine 5–10 mg/kg or telazol 3–6 mg/kg) and analgesics for IM immunizations, LN FNA, bone marrow aspirates, and blood draws as per veterinarian recommendations and IACUC-approved protocols. After completion of the proposed study and approval of the vet staff, animals were released to the center for reuse by other researchers.

### ELISA

On Day 1, human PGT128 capture antibody was coated onto ELISA plates (Corning 96-Well Half-Area Plates, Catalog #3690) at a concentration of 1 or 2 μg/mL in 25 μl PBS (pH 7.4; Thermo Scientific, Catalog #10010–023) per well. Plates were incubated overnight at 4°C. On day 2, plates were washed three times with PBST (PBS + 0.2% Tween-20) and blocked for 1 hour at room temperature with PBST containing 5% skim milk (BD Difco Skim Milk, Catalog #232100) and 1% FBS (Thermo Fisher, Catalog #16000044). After blocking, plates were washed three times with PBST, and 25 μl of ELISA antigen (0.3, 1, or 2 μg/mL) was added per well. Plates were incubated for 2 hours at room temperature, followed by three washes with PBST. Subsequently, serum serially diluted (starting at 1:100, 3-fold dilutions) in blocking buffer (PBST + 1% FBS) was added and incubated for 1 hour at 37°C with 80% humidity. Plates were washed three times, and 25 μl of secondary antibody was added as follows: for rabbit serum, Peroxidase AffiniPure Donkey Anti-Rabbit IgG (H+L) (Jackson ImmunoResearch, Catalog #711–035-152), diluted 1:5,000 in PBST + 1% FBS was used; for NHP serum, Peroxidase AffiniPure Donkey Anti-Human IgG, Fcγ fragment specific (Jackson ImmunoResearch, Catalog #709–035-098), diluted 1:5,000 in PBST + 1% FBS was used. Plates were incubated for 1 hour at room temperature, washed three times, and developed using TMB Chromogen Solution (Thermo Fisher, Catalog #002023) diluted 1:4. After 6 minutes, the reaction was stopped by adding 25 μl of 0.5 M H₂SO₄. Absorbance was measured at 450 nm and 570 nm using a BioTek Epoch 2 Microplate Reader (BioTek). Background subtraction was performed by subtracting the 570 nm value from the corresponding 450 nm value. The resulting data were analyzed using Prism software (v10.4.1; GraphPad) to calculate the area under the curve (AUC) and the reciprocal dilution of serum at which 50% of the maximum binding response was observed (ED_50_). AUC values were determined using the trapezoidal method, which connects adjacent points with straight lines and sums the areas under these segments to approximate the total curve area. ED_50_ values were calculated using Agonist vs response equation.

### Neutralization assay

Pseudovirus neutralization assays were performed as previously described using the BG505 T332N pseudovirus (54). Single-cycle infectious pseudoviruses were generated by co-transfecting HEK293T cells with the envelope (Env) proteins of interest and the Env-deficient HIV-1 backbone plasmid pSG3ΔEnv, using either Fugene 6 (Promega, E2692) or PEI MAX (Polysciences, 24765–1). Viral supernatants were collected 72 hours post-transfection and stored at –80°C until use.

For the assay, TZM-bl target cells were seeded the day prior at a density of 100,000 cells/mL in 50 εL volumes into half-area 96-well plates (Corning, 3688). On the assay day, monoclonal antibodies (mAbs) were serially diluted in D10 media, composed of DMEM (Gibco, 10313–021), 10% FBS (Omega Scientific, FB-02), 1× Penicillin-Streptomycin (Gibco, 15070–063), and 1× GlutaMAX (Gibco, 35050–061). Pseudoviruses were thawed at 37°C and adjusted to the desired concentration using D10 medium, based on pre-determined titers. DEAE-dextran (Spectrum Chemical, DE132) was added to the viral preparations at a final concentration of 10 εg/mL. Equal volumes (1:1, v/v) of virus and diluted mAbs were combined in round-bottom 96-well plates (Corning, 3788) and incubated at 37°C for 1 hour. After incubation, the media from the TZM-bl cell plates was carefully aspirated, and 25 εL of the virus–mAb mixture was added to each well. Plates were returned to the incubator, and after 24 hours, 75 εL of D10 media was added to each well. Cells were incubated for an additional 48 hours. Following the incubation period, the supernatant was removed, and cells were lysed with 45 εL/well of 1× Cell Lysis Buffer (Promega, E4550) for 15 minutes. Then, 30 εL/well of luciferase substrate (Promega, E4550) was added, and luminescence was measured using a BioTek Synergy H1 Plate Reader. Half-maximal inhibitory concentrations (IC_50_) were determined using the “One site – Fit logIC50” model in GraphPad Prism 9, with all curve fits constrained between 0 and 100% neutralization. Both TZM-bl and HEK293T cells were maintained in D10 media under standard conditions (37°C, humidified incubator with 5% CO₂).

### EMPEM

Polyclonal Fabs were prepared as previously described (55). Briefly, IgG was isolated from heat-inactivated serum samples using a HiTrap MAbSelect PrismA protein A column (Cytiva). Purified polyclonal IgG was digested using papain to generate polyclonal Fab, and Fc and undigested IgG was removed by incubating with CaptureSelect IgG-Fc multispecies affinity resin (Thermo Fisher). 0.5 mg of each polyclonal Fab sample was complexed with 15 εg of either BG505 MD39.3 or 15 εg of an 80/20 mixture of BG505 MD39.3 CC5 (containing 2 engineered disulfides: 49C-555C, 73C-561C) and BG505 MD39.3. Samples were incubated overnight at 20°C. Complexes were purified over a Superdex 200 Increase size exclusion column (Cytiva). EM grids were prepared by diluting complexes were to approximately 0.03 mg/mL (based on trimer concentration) in 1X TBS. A 3 εL drop of sample was applied to a glow-discharged carbon-coated copper mesh grid for 10 seconds, blotted with Whatman #1 filter paper, and a 3 εl drop of 2% (w/v) uranyl formate solution was applied for 45 to 60 seconds, followed by blotting. Grids were imaged using an FEI Tecnai Spirit microscope operating at 120 keV, equipped with a TVIPS TemCam F416 camera, and 52,000x magnification (resulting in a 2.06 Å pixel size). Data acquisition was automated using Leginon (56) and processed using Relion 4.0 (52). Visualization and image generation was performed using UCSF ChimeraX (57). For a subset of samples, EMPEM was repeated using the MD39.3 BaseKO antigen following the methods described above. Representative EM maps have been deposited to the Electron Microscopy Data Bank (EMDB) under accession codes EMD-70838, EMD-70839, EMD-70840, EMD-70846, EMD-70847, EMD-70848, EMD-70852, EMD-70855, EMD-70858 and EMD-70860.

### Flow cytometry

Cryopreserved PBMCs or LN FNAs were thawed and washed in R10 media made with RPMI (Corning, Catalog #10–041-CV) supplemented with 10% FBS (GeminiBio, Catalog #900–108), 1% Penicillin-Streptomycin (Thermo Fisher Scientific, Catalog #15140–122) and 1% GlutaMax (Thermo Fisher Scientific, Catalog #35050–061). Tetramerized fluorescent probes for MD39.3 and MD39.3 CD4KO and corresponding BaseKO were prepared by mixing each biotinylated probe with streptavidin-conjugated fluorophores in stepwise addition and incubated in the dark at room temperature for a total of 45 minutes. MD39.3 BaseKO tetramers were added first to the cells and incubated for 20 minutes on ice, followed by addition of MD39.3 tetramers for 30 minutes and lastly adding the complete surface antibody staining cocktail for 30 minutes on ice. Stained cells were washed and analyzed on an Aurora flow cytometer (Cytek Biosciences) or sorted on the FACSymphony S6 (BD Biosciences). Anti-CD38–PE-Cy5 was conjugated using purified anti-CD38 (OKT10; Nonhuman Primate Reagent Resource [NHPRR] PR-0056) and the PE/Cy5 Conjugation Kit (Abcam, Catalog #ab102893). Sorted week 24 samples (group 2 & 5) analysis was included with other sample data analyzed on the Aurora flow cytometer and included in Fig. 2D for longitudinal responses. Antibody panels are summarized in table S2 to S4. For single cell sequencing analysis, TotalSeq-C anti-human Hashtag antibodies were used to multiplex samples. TotalSeq-C phycoerythrin (PE)-streptavidin and custom TotalSeq-C brilliant violet (BV) 421-streptavidin conjugates were used for probe labeling (BioLegend). The limit of detection (LOD) for Env-binding B_GC_ cells was calculated based on the median of (3/(number of B cells collected)) from the LN FNA samples at the pre-immunization timepoint and for Env-binding B_Mem_ cells was calculated based on the median from the PBMC samples at the pre-immunization timepoint.

### Antigen-specific T cell AIM and ICS assays

Cryopreserved PBMCs were thawed and washed in R10 media. Cells were counted and seeded at 1 × 10^6^ per well in a round-bottom 96-well plate in presence of 5 εg/mL MD39.3 peptide pool; 1 ng/mL staphylococcal enterotoxin B (SEB) was used as a positive control and DMSO was used as a negative control (an equimolar amount of DMSO is present in the peptide pool, which was plated in duplicate). Prior to addition of peptides, cells were blocked with 0.5 μg/mL anti-CD40 mAb (Miltenyi Biotec, Catalog # 130–094-133) and incubated with anti-CXCR5 (1:100, Thermo Fisher Scientific, Catalog #25–9185-42) and anti-CCR7 (1:100, BioLegend, Catalog #353233) 15 min at 37° C. After 24 hours of incubation, intracellular transport inhibitors (0.25 μl/well of GolgiStop (BD Biosciences, Catalog #554724) and GolgiPlug (BD Biosciences, Catalog #555029)). Next, the AIM marker antibodies were added to the samples and incubated for an additional 4 hours. At the end of the culture, cells were washed and stained with surface markers for 30 minutes at 4°C before fixation and permeabilization and subsequent intracellular cytokine staining at room temperature for 30 minutes. Stained cells were washed and analyzed on an Aurora flow cytometer (Cytek Biosciences). Antibody panels are summarized in table S5. Antigen-specific CD4^+^ and CD8^+^ T cells were calculated by subtracting the background data (average DMSO response). A minimum threshold for DMSO-stimulated cell frequencies was set at 0.005%. For each sample, the stimulation index (SI) is calculated as the ratio of the frequency of AIM^+^ cells in the MD39.3 stimulated condition compared with the averaged DMSO response for the same sample. The limit of quantification (LOQ) was set at the geometric mean of all DMSO samples. Samples with an SI < 2 for CD4^+^ T cell or 3 for CD8^+^ T cell responses or with a background-subtracted response <LOQ were considered non-responders. Non-responder samples were set at the baseline value, defined as 2-fold below the LOQ.

### B cell receptor sequencing and analysis

Env-binding B_Mem_ cells from NHPs in the soluble mRNA and protein immunization groups were sorted at various time points (weeks 8, 10, 24, 26 and 32) for BCR sequencing. Sorted Env-binding B_Mem_ cells, Single Cell VDJ 5’ Gel Beads and partitioning oil were loaded into the designated wells of the Chromium Next GEM Chip K and run on the Chromium Controller for the generation of single cell droplets. GEX, VDJ and Feature Barcode libraries were generated using the Chromium Next GEM Single Cell 5’ Reagent Kits v2 or v3 (10X Genomics) according to manufacturer’s recommendations. Custom primers specific for NHP immunoglobulin constant regions were designed for the VDJ amplification step (table S6). Samples were sequenced on the NovaSeq 6000 (Illumina). Raw sequences were assembled using the de novo option in CellRanger (10X Genomics). Assembled contigs were annotated using IgBLAST on a custom rhesus macaque reference and further processing was done using the Change-O pipeline from the Immcantation portal (58). The SHazaM package was used to determine mutations in the BCR sequences; clonally-related sequences were clustered with DefineClones.py using the appropriate threshold. Sequences with IgM or NA isotypes were excluded from the SHM analysis.

### SPR

We measured kinetics and affinity of antibody-antigen interactions on a Carterra LSA. This was done with a low-capture IgG method as previously described (48). Epitope mapping was performed using the knockout versions of BG505 MD39.3.

### Single cell transcriptomics analysis

Transcriptomic analyses were conducted on the same sorted Env-binding B_Mem_ from the soluble mRNA and protein immunization groups used for BCR sequencing from various timepoints (weeks 8, 10, 24, 26 and 32) in NHPs to evaluate potential differences within these populations. Animals were randomized prior to immunization and baseline responses were expected to be comparable across groups. Transcriptomic analysis was conducted using Seurat (v5.0.1). Hashtag oligos were demultiplexed using the MULTIseqDemux function to assign sequences to the corresponding animals. Quality control measures excluded cells with fewer than 200 or more than 4,500 features, as well as those with mitochondrial RNA percentages exceeding 5%. Doublets and non-B cells were removed from the analysis. Datasets were then integrated using the anchor-based canonical correlation analysis (CCA) method. Further, Ig (LOC-) and MHC (MAMU-) genes were ignored for clustering.

### Statistical Analysis

Individual-level data for experiments where n<20 are presented in data file S1. Statistical analyses were conducted using Prism v10 (GraphPad Software). Prior to the start of the study, we hypothesized that a greater number of base-binding antibodies would be induced in the soluble mRNA group compared with the membrane-bound mRNA group. For statistical analyses based on this predefined hypothesis or for comparisons involving only two groups at one timepoint, an unpaired Mann-Whitney test was used. For pairwise comparisons between two groups across multiple time points, the Mann-Whitney test with Holm-Šídák correction for multiple comparisons was applied. For comparisons among multiple groups, the Kruskal-Wallis test followed by Dunn’s test for multiple comparisons was used. When tests were limited to specific groups or time points, this was indicated in the figure legends or main text.

## Supplementary Material

Supplementary Materials

Data file S1

MDAR Reproducibility Checklist

Fig. S1 to S7

Tables S1 to S6

MDAR Reproducibility Checklist

Data file S1

## Data Availability

All data associated with this study are included in the paper or the Supplementary Materials. Plasmids or proteins related to the immunogens, sort reagents, or antibodies used in this study are available from W.R.S. (schief@scripps.edu) under a material transfer agreement with the Scripps Research Institute. mRNA vaccine constructs can be made available from S.H. (Sunny.Himansu@modernatx.com) under a material transfer agreement with Moderna. The scRNA-seq data have been deposited in the GEO database under accession number GSE298795. Representative EMPEM maps have been deposited to the Electron Microscopy Data Bank (EMDB) under accession codes EMD-70838, EMD-70839, EMD-70840, EMD-70846, EMD-70847, EMD-70848, EMD-70852, EMD-70855, EMD-70858 and EMD-70860.
