# Supplementary material for: Vaccination with an mRNA-encoded membrane-bound HIV Envelope trimer induces neutralizing antibodies in animal models
Source: Sci Transl Med. Author manuscript; Available in PMC 2025 Aug 19. (PMC12363394; doi:10.1126/scitranslmed.adw0721)
Supplement: Supplementary Materials [file NIHMS2100104-supplement-Supplementary_Materials.pdf]

A

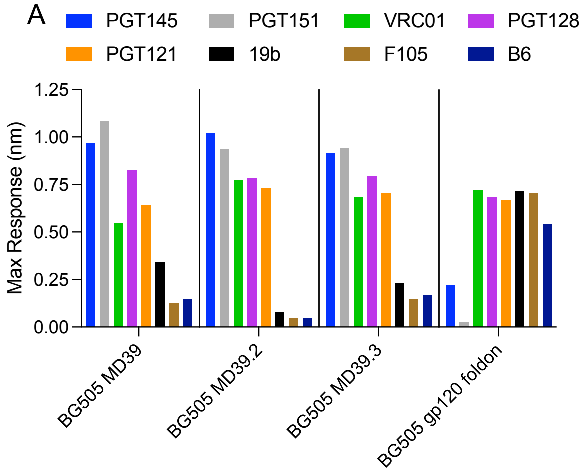

B

Name	Post SEC Yield (mg/L)	Tm (°C)
BG505 MD39 gp140	3.6	78.7
BG505 MD39.2 gp140	3.3	73.6
BG505 MD39.3 gp140	3.1	75.6

C

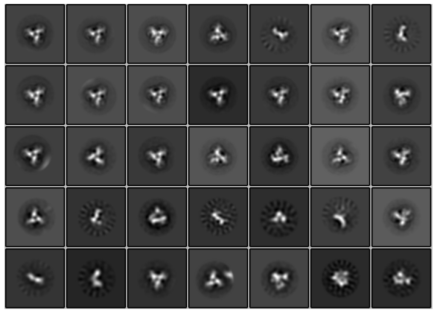

82% native-like trimers

D

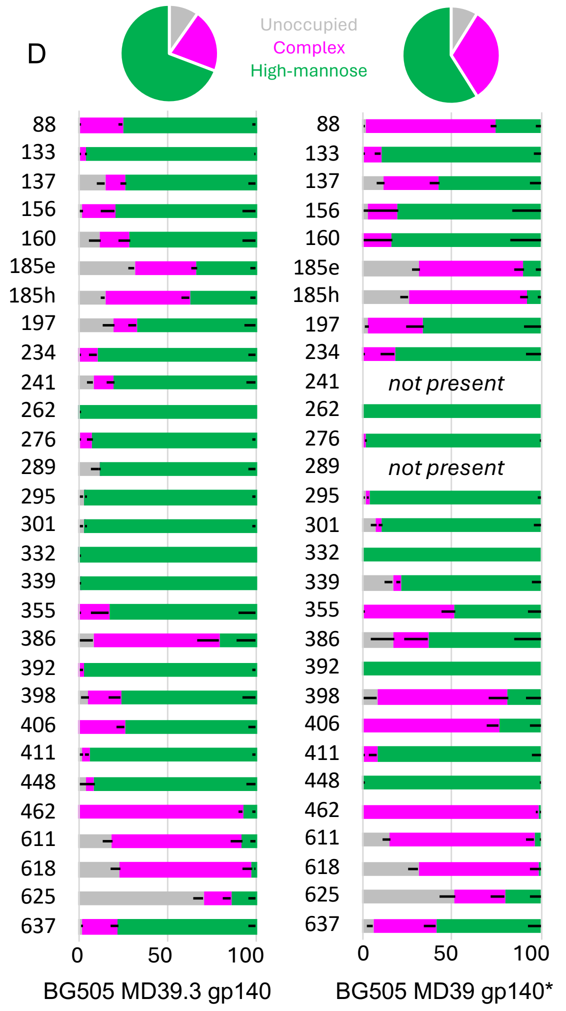

E

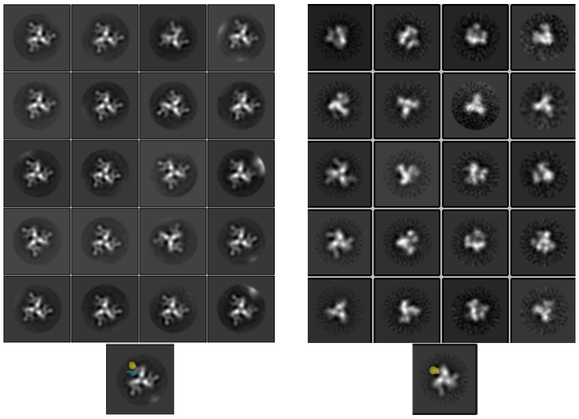

F

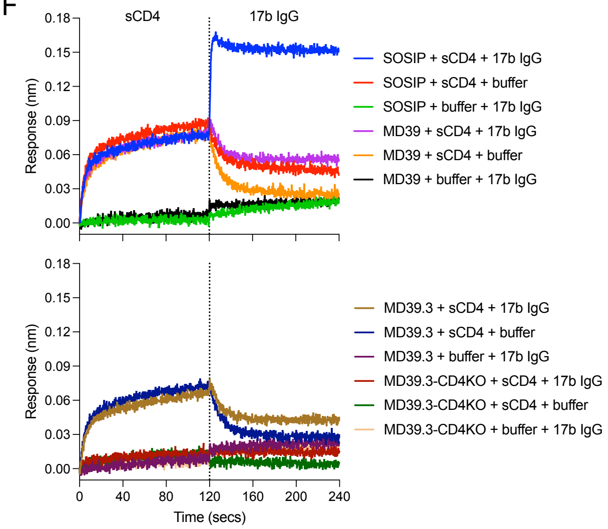

Figure S1

Fig. S1. Antigenic profiling, biophysical characterization, and structural analysis of BG505-based HIV-1 trimer immunogens. (**A**) Biolayer interferometry (BLI) was used to assess antigenic profiles for the indicated trimers binding to IgG broadly neutralizing antibodies (bnAbs; quaternary, PGT151 and PGT145; CD4bs, VRC01; and V3-glycan, PGT121 and PGT128) and non-nAbs (V3, 19b; CD4bs, B6 and F105). (**B**) Yield and thermostability of BG505 MD39 based immunogens. Yield was determined after 2G12 affinity chromatography and size exclusion chromatography (SEC) purification. Thermostability measurements (Tm) were made using nano differential scanning fluorimetry. (**C**) Negative stain electron microscopy analysis of BG505 MD39.3 gp140. (**D**) Glycan analysis of BG505 MD39 gp140 and BG505 MD39.3 gp140. Green indicates high mannose glycans, pink indicates complex type glycans, gray indicates unoccupied glycosylation sites, and black bars indicate standard error of the mean. The N241 and N289 glycosylation sites are not present on BG505 MD39 gp140. Glycosylation sites are numbered using HxB2 numbering. *Adapted with permission from (*53*). (**E**) Negative stain electron microscopy analysis of BG505 SOSIP gp140 or BG505 MD39 gp140 with human sCD4. Example class averages with sCD4 and the exposed V1/V2 are highlighted in yellow and blue, respectively, for reference. (**F**) Binding of sCD4 followed by 17b IgG for BG505 SOSIP, BG505 MD39, BG505 MD39.3, and BG505 MD39.3-CD4KO assessed by BLI.

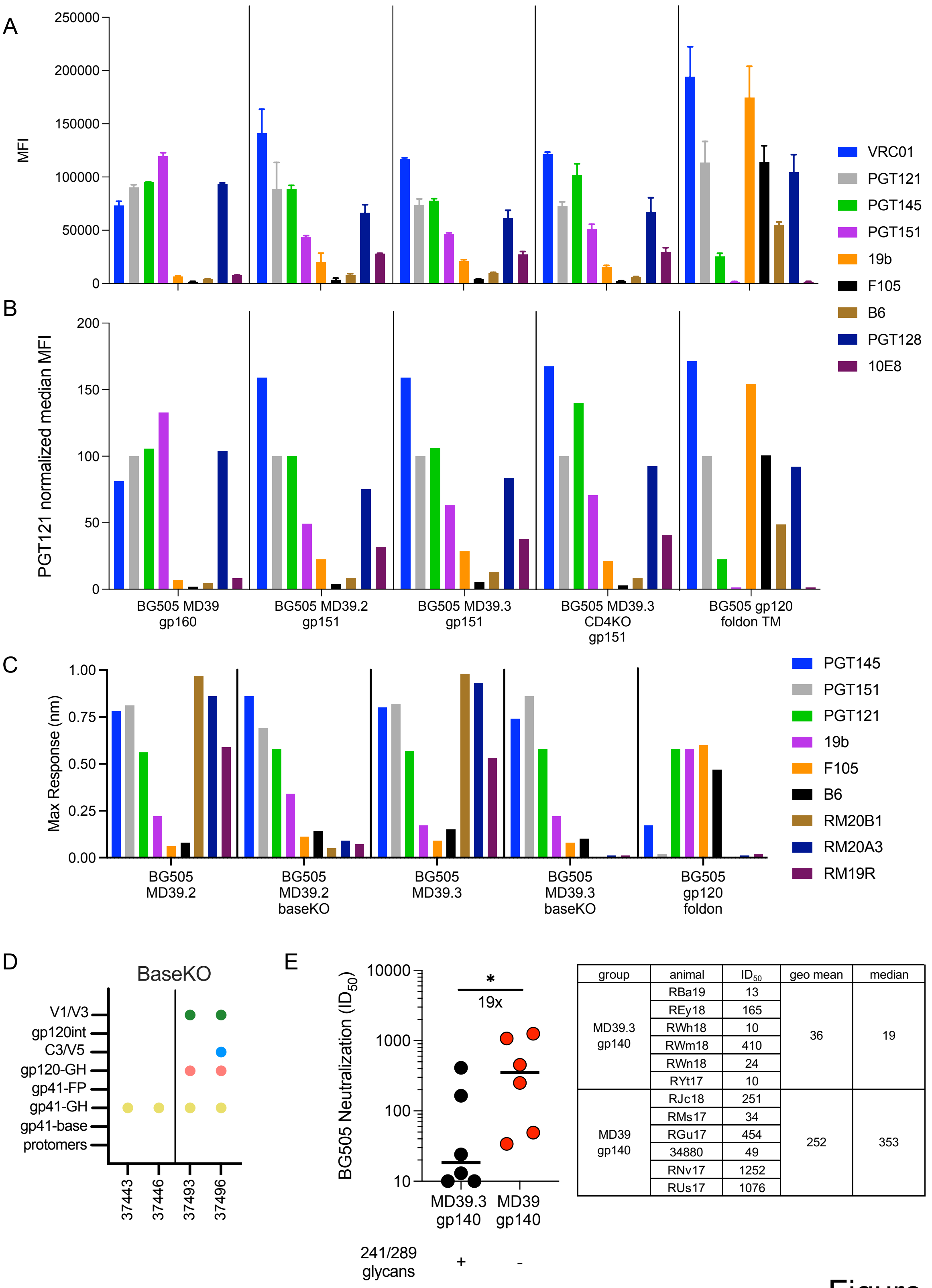

group	animal	ID_{50}	geo mean	median
MD39.3 gp140	RBa19	13	36	19
	REy18	165		
	RWh18	10		
	RWm18	410		
	RWn18	24		
	RYt17	10		
MD39 gp140	RJc18	251	252	353
	RMs17	34		
	RGu17	454		
	34880	49		
	RNv17	1252		
	RUs17	1076		

Figure S2

Fig. S2. Cell surface antigenicity and BaseKO validation. Flow cytometry analysis of HEK293F cells transfected with DNA plasmids encoding membrane-bound HIV Env constructs. Cells were stained with bnAbs that were quaternary specific (PGT145 and PGT151), CD4bs specific (VRC01), V3-glycan specific (PGT121 and PGT128), MPER specific (10E8), or with non-neutralizing antibodies (F105, B6, and 19b). (**A**) Raw mean fluorescence intensity (MFI) values show expression of each construct. Median values plotted with error bars showing the range (n=2). (**B**) PGT121 normalized MFI values showing antigenicity scaled to expression for each construct. (**C**) BLI antigenic profiles of MD39.2 BaseKO and MD39.3 BaseKO along with their parental antigens. (**D**) Electron microscopy polyclonal epitope mapping (EMPEM) analysis using MD39.3 BaseKO antigen. (**E**) Comparison of effect of glycan hole for autologous neutralization. Week 26 serum neutralization titers against BG505 T332N pseudovirus using samples from MD39.3 gp140 protein (G5)-vaccinated non-human primates (NHPs) were compared with samples from glycan hole-containing MD39 gp140 protein-vaccinated NHPs reported in Silva *et al.* (*22*). ID_{50}, half-maximal inhibitory dilution. Geo mean, geometric mean. Bars indicate median for neutralization data. Statistical significance was assessed using the Mann-Whitney test. Significance levels are indicated as $^*P < 0.05$.

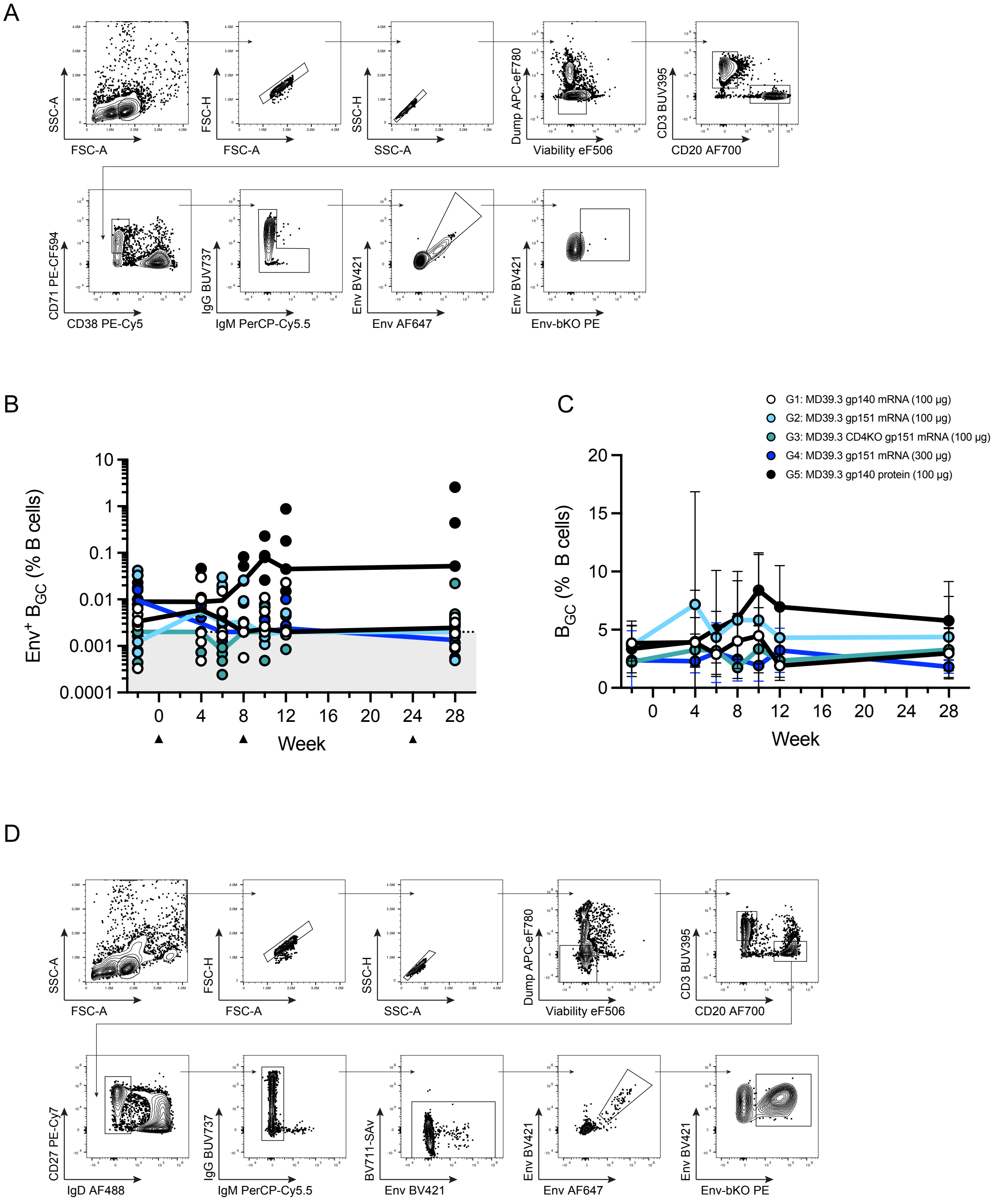
A
SSC-A
FSC-A
FSC-H
FSC-A
SSC-H
SSC-A
Dump APC-eF780
Viability eF506
CD3 BUV395
CD20 AF700
CD71 PE-CF594
CD38 PE-Cy5
IgG BUV737
IgM PerCP-Cy5.5
Env BV421
Env AF647
Env BV421
Env-bKO PE
B
Env+ BGC (% B cells)
10
1
0.1
0.01
0.001
0.0001
0
4
8
12
16
20
24
28
Week
C
G1: MD39.3 gp140 mRNA (100 μg)
G2: MD39.3 gp151 mRNA (100 μg)
G3: MD39.3 CD4KO gp151 mRNA (100 μg)
G4: MD39.3 gp151 mRNA (300 μg)
G5: MD39.3 gp140 protein (100 μg)
BGC (% B cells)
20
15
10
5
0
0
4
8
12
16
20
24
28
Week
D
SSC-A
FSC-A
FSC-H
FSC-A
SSC-H
SSC-A
Dump APC-eF780
Viability eF506
CD3 BUV395
CD20 AF700
CD27 PE-Cy7
IgD AF488
IgG BUV737
IgM PerCP-Cy5.5
BV711-SAv
Env BV421
Env BV421
Env AF647
Env BV421
Env-bKO PE

Figure S3

Fig. S3. Gating strategy for germinal center B cells (B_{GC} cells) in lymph nodes (LNs) and memory B cells (B_{Mem} cells) in peripheral blood mononuclear cells (PBMCs) from vaccinated NHPs. (**A**) Flow cytometry gating strategy of Env-binding B_{GC} cells in LN fine needle aspirates (FNAs). (**B**) Frequency of Env-binding B_{GC} cells in sampled LNs across groups and timepoints. Arrowheads indicate week of vaccination. (**C**) Frequency of total B_{GC} cells in sampled LNs across groups and timepoints. (**D**) Flow cytometry gating strategy of Env-binding B_{Mem} cells in PBMCs. In (B), each point indicates a single animal (n=6 per group), the curves indicate the median and the gray shading represents the LOD. Each point indicates the mean with corresponding error in (C). Animals with less than 75 B_{GC} cells were excluded from the analysis.

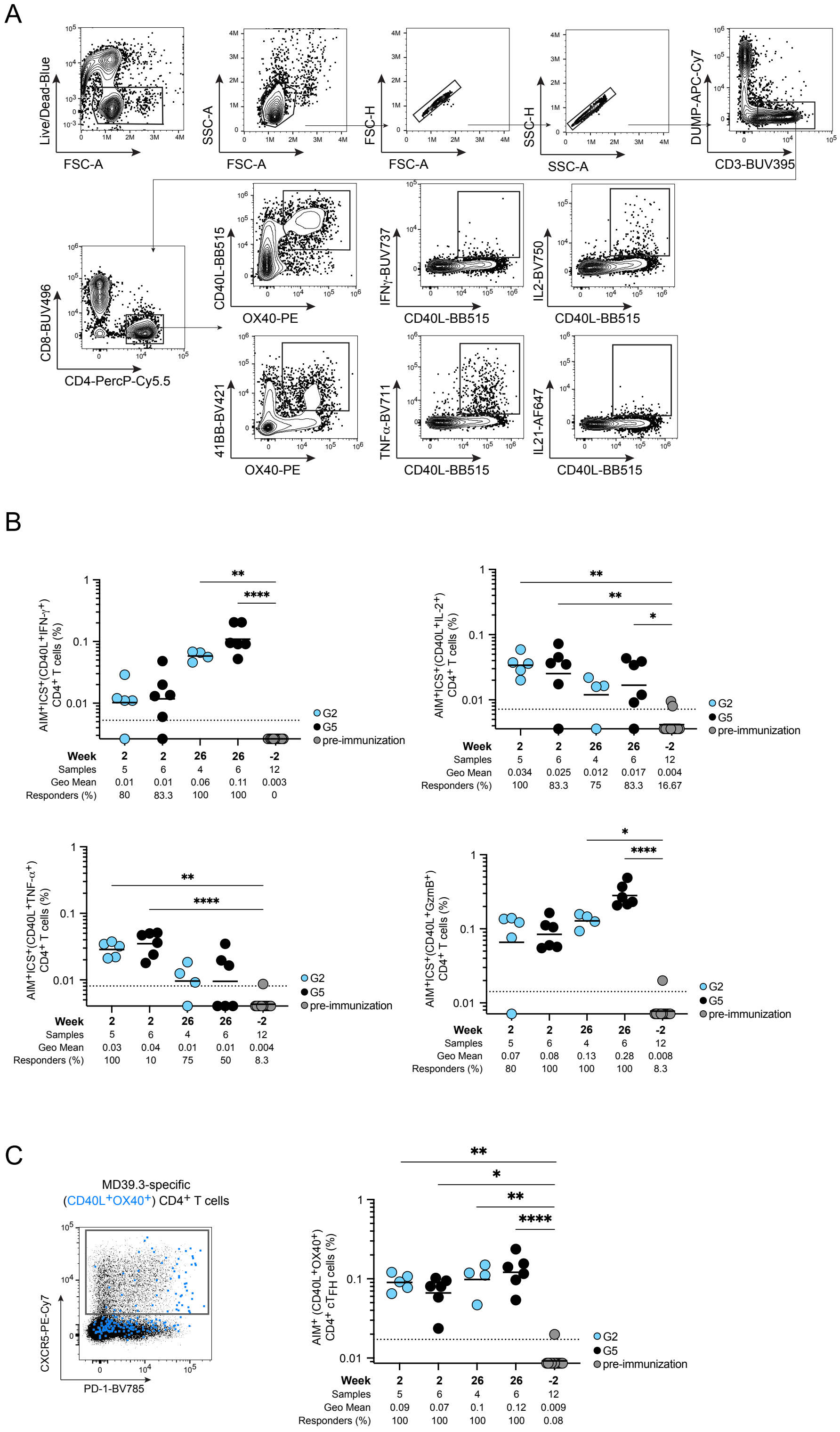
A
Live/Dead-Blue
FSC-A
SSC-A
FSC-A
FSC-H
FSC-A
SSC-H
SSC-A
DUMP-APC-Cy7
CD3-BUV395
CD8-BUV496
CD4-PercP-Cy5.5
CD40L-BB515
OX40-PE
IFNγ-BUV737
CD40L-BB515
IL2-BV750
CD40L-BB515
41BB-BV421
OX40-PE
TNFα-BV711
CD40L-BB515
IL21-AF647
CD40L-BB515
B
AIM+ICS+(CD40L+IFN-γ+) CD4+ T cells (%)
Week 2 2 26 26 -2
Samples 5 6 4 6 12
Geo Mean 0.01 0.01 0.06 0.11 0.003
Responders (%) 80 83.3 100 100 0
AIM+ICS+(CD40L+IL-2+) CD4+ T cells (%)
Week 2 2 26 26 -2
Samples 5 6 4 6 12
Geo Mean 0.034 0.025 0.012 0.017 0.004
Responders (%) 100 83.3 75 83.3 16.67
AIM+ICS+(CD40L+TNF-α+) CD4+ T cells (%)
Week 2 2 26 26 -2
Samples 5 6 4 6 12
Geo Mean 0.03 0.04 0.01 0.01 0.004
Responders (%) 100 10 75 50 8.3
AIM+ICS+(CD40L+GzmB+) CD4+ T cells (%)
Week 2 2 26 26 -2
Samples 5 6 4 6 12
Geo Mean 0.07 0.08 0.13 0.28 0.008
Responders (%) 80 100 100 100 8.3
G2
G5
pre-immunization
C
MD39.3-specific (CD40L+OX40+) CD4+ T cells
CXCR5-PE-Cy7
PD-1-BV785
AIM+ (CD40L+OX40+) CD4+ cTFH cells (%)
Week 2 2 26 26 -2
Samples 5 6 4 6 12
Geo Mean 0.09 0.07 0.1 0.12 0.009
Responders (%) 100 100 100 100 0.08
G2
G5
pre-immunization

Figure S4

Fig. S4. Activation-induced marker (AIM) and intracellular cytokine staining (ICS) T cell assays and gating strategy. (**A**) Flow cytometry gating strategy of AIM and ICS assays to detect antigen-specific $CD4^+$ T cells. (**B**) Quantification of Env-specific cytokine-producing (AIM^+ICS^+) $CD4^+$ T cells are shown as a percentage of $CD4^+$ T cells for G2 and G5 at week 2 (post-prime) and week 26 (post-third dose). IFN-γ, interferon-γ; TNF-α, tumor necrosis factor-α; IL-2, interleukin-2; GzmB, granzyme B. (**C**) Shown are representative flow plots and quantification of Env-specific $CXCR5^+PD\text{-}1^{+/-}$ circulating T follicular helper (cT_{FH}) cells as a percentage of $CD4^+$ T cells for G2 and G5 at week 2 (post-prime) and week 26 (post-third dose). Dots highlighted in blue represent antigen-specific $CD4^+$ T cells ($CD40L^+OX40^+$). For (B) and (C), data are shown as background subtracted. Non-responder samples are set at baseline. The dotted black line indicates the limit of quantification (LOQ). Bars represent geometric mean and each point indicate a single animal (n=6 per group). The number of samples per group, the geometric mean (geo mean) frequency, and the frequency of responders per group is shown below each plot. Animals with missing or poor-quality samples were excluded from the analysis. Statistical significance was assessed using the Kruskal-Wallis test, followed by Dunn's multiple comparisons test. Significance levels are indicated as $*P < 0.05$, $**P < 0.01$, and $****P < 0.0001$.

A

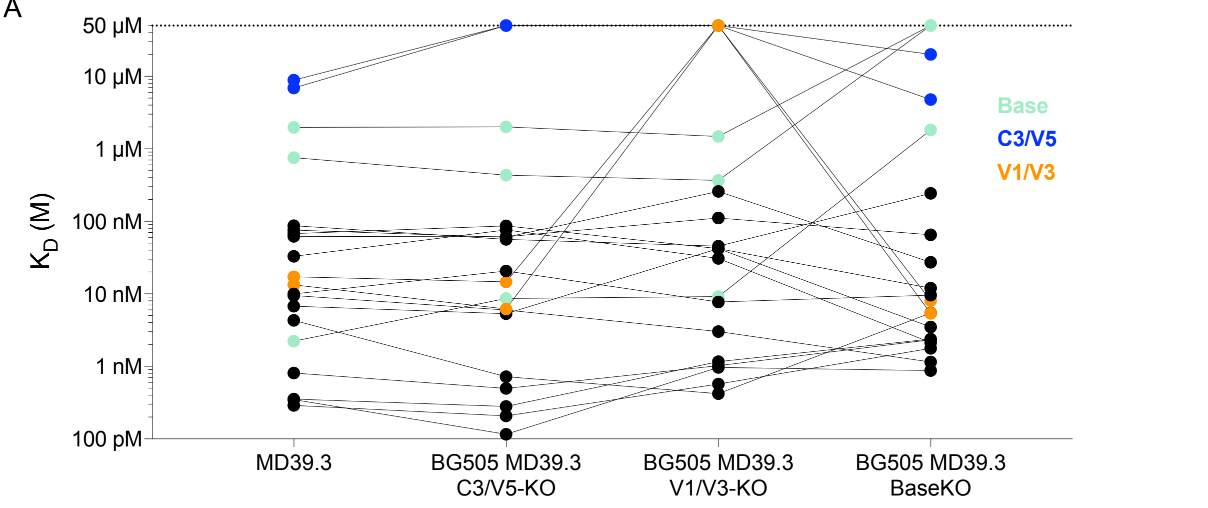

B

	BG505 WT	BG505 T332N	BG505 N611A	BG505 N241+N289	BG505 133aN	BG505 133aN+136aA	BG505 T465N	MLV
VRC01	0.084	0.047	0.089	0.050	0.120	0.091	0.084	NN
RFu18_1_IgG	NN	NN	NN	NN	NN	NN	NN	NN
RFu18_10_IgG	NN	NN	NN	NN	NN	NN	NN	NN
RFu18_12_IgG	NN	NN	NN	NN	NN	NN	NN	NN
RFu18_2_IgG	NN	NN	NN	NN	NN	NN	NN	NN
RFu18_4_IgG	NN	NN	NN	NN	NN	NN	NN	NN
RFu18_6_IgG	NN	NN	NN	NN	NN	NN	NN	NN
RFu18_37_IgG	NN	NN	NN	NN	NN	NN	NN	NN
RFu18_38_IgG	NN	NN	NN	NN	NN	NN	NN	NN
RUv18_29_IgG	NN	NN	NN	NN	NN	NN	NN	NN
RUv18_30_IgG	NN	NN	NN	NN	NN	NN	NN	NN
RUv18_31_IgG	NN	NN	NN	NN	NN	NN	NN	NN
RUv18_40_IgG	NN	NN	NN	NN	NN	NN	NN	NN
RUv18_42_IgG	NN	NN	NN	NN	NN	NN	NN	NN
RUv18_43_IgG	1.477	4.154	1.072	12.911	NN	NN	13.557	NN
RFu18_11_IgG	NN	21.955	NN	NN	NN	NN	NN	NN
RUv18_44_IgG	0.094	0.549	0.360	0.730	NN	NN	0.960	NN
RUv18_46_IgG	NN	NN	NN	NN	NN	NN	NN	NN
RUv18_47_IgG	NN	NN	NN	NN	NN	NN	NN	NN
RWo17_32_IgG	NN	NN	NN	NN	NN	NN	NN	NN
DEN	NN	NN	NN	NN	NN	NN	NN	NN

IC_{50} (µg/mL)
0.001-0.010
0.010-0.100
0.100-1.000
1.000-10.00
10.00-50.00

C

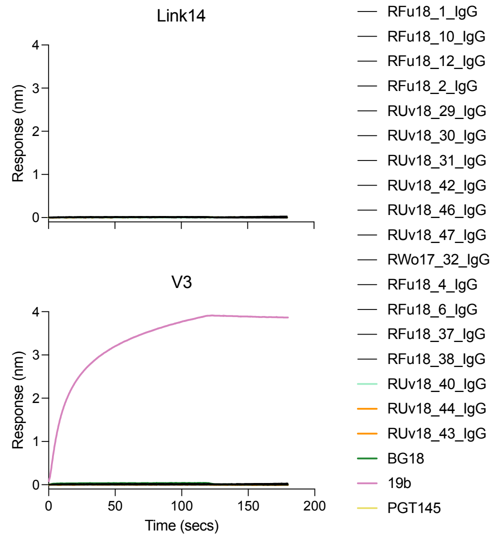

Figure S5

Fig. S5. Characterization of monoclonal antibodies (mAbs) derived from Env-binding B_{Mem} cells in vaccinated NHPs. (**A**) Shown are surface plasmon resonance (SPR) data of mAb affinities and binding to different MD39.3 proteins with mAbs targeting the base epitope in green, the C3/V5 epitope in blue, and the V1/V3 epitope in orange. mAbs targeting unknown epitopes are shown in black. K_D, dissociation constant. (**B**) Neutralization of mAbs tested for a panel of different viruses with the same coloring as (A); IC_{50}, half-maximal inhibitory concentration; NN, no neutralization; DEN, dengue virus nonstructural protein 1 specific antibody DEN3 used as a negative control; MLV, Murine Leukemia Virus. (**C**) Binding of mAbs to Link14 and V3 peptides was assessed by BLI with the same coloring as (A).

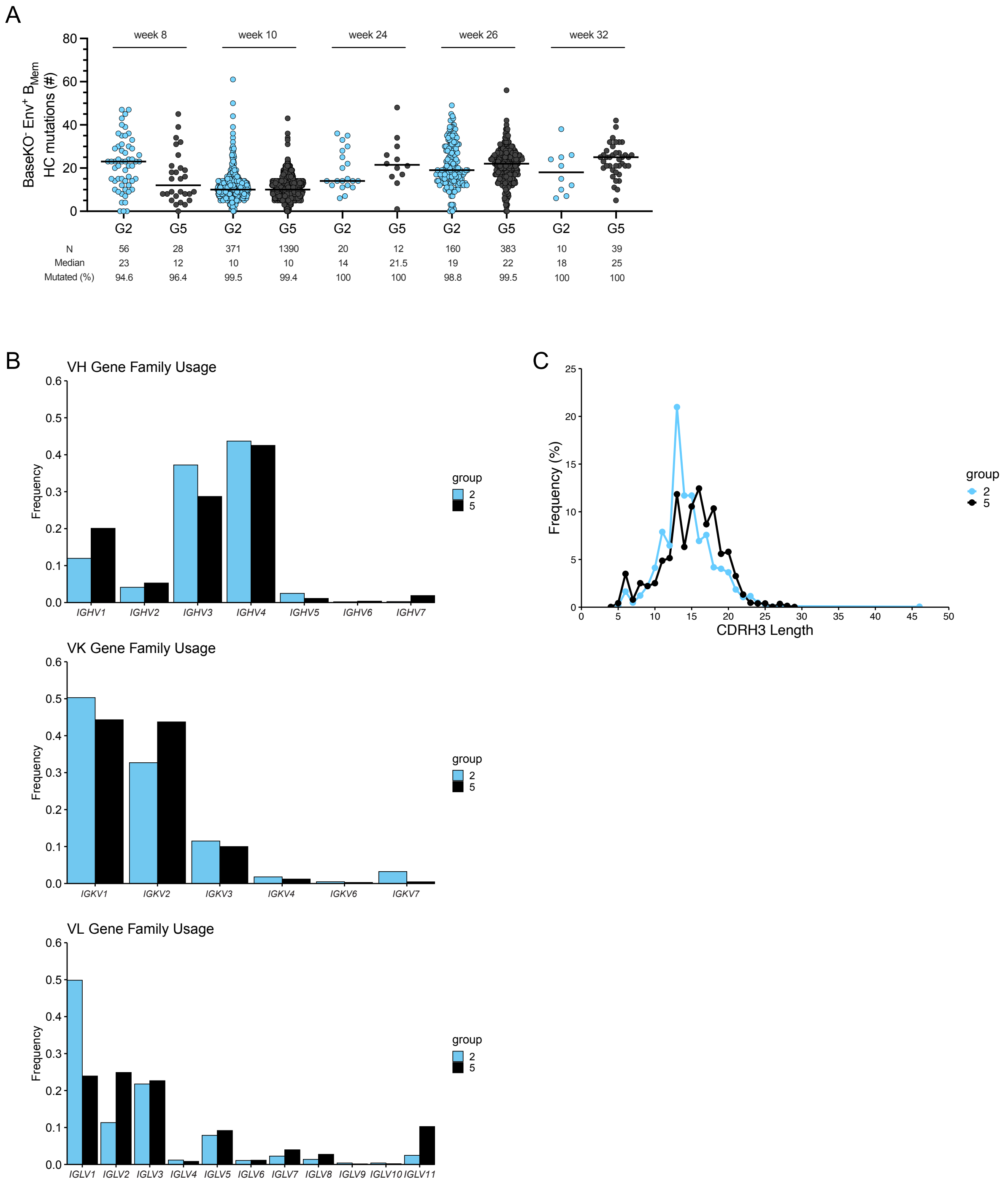
A
week 8
week 10
week 24
week 26
week 32
BaseKO- Env+ BMem HC mutations (#)
G2 G5 G2 G5 G2 G5 G2 G5 G2 G5
N 56 28 371 1390 20 12 160 383 10 39
Median 23 12 10 10 14 21.5 19 22 18 25
Mutated (%) 94.6 96.4 99.5 99.4 100 100 98.8 99.5 100 100
B
VH Gene Family Usage
Frequency
IGHV1 IGHV2 IGHV3 IGHV4 IGHV5 IGHV6 IGHV7
group
2
5
VK Gene Family Usage
IGKV1 IGKV2 IGKV3 IGKV4 IGKV6 IGKV7
VL Gene Family Usage
IGLV1 IGLV2 IGLV3 IGLV4 IGLV5 IGLV6 IGLV7 IGLV8 IGLV9 IGLV10 IGLV11
C
Frequency (%)
CDRH3 Length
group
2
5

Figure S6

Fig. S6. V gene usage of Env-binding B_{Mem} cells in NHPs. (**A**) Heavy chain mutations in base-Env-binding B_{Mem} cells (BaseKO^{-}) were assessed for G2 and G5 at week 8, 10, 24, 26 and 32. The number of B cell sequences analyzed, the median number of HC mutations, and the frequency of mutated HCs are shown below each plot. (**B**) Shown is V gene usage of heavy and light (kappa and lambda) chains of sequenced Env-binding B_{Mem} cells from G2 and G5. (**C**) Shown are heavy chain complementarity determining region 3 (CDRH3) lengths of sequenced Env-binding B_{Mem} cells from G2 and G5.

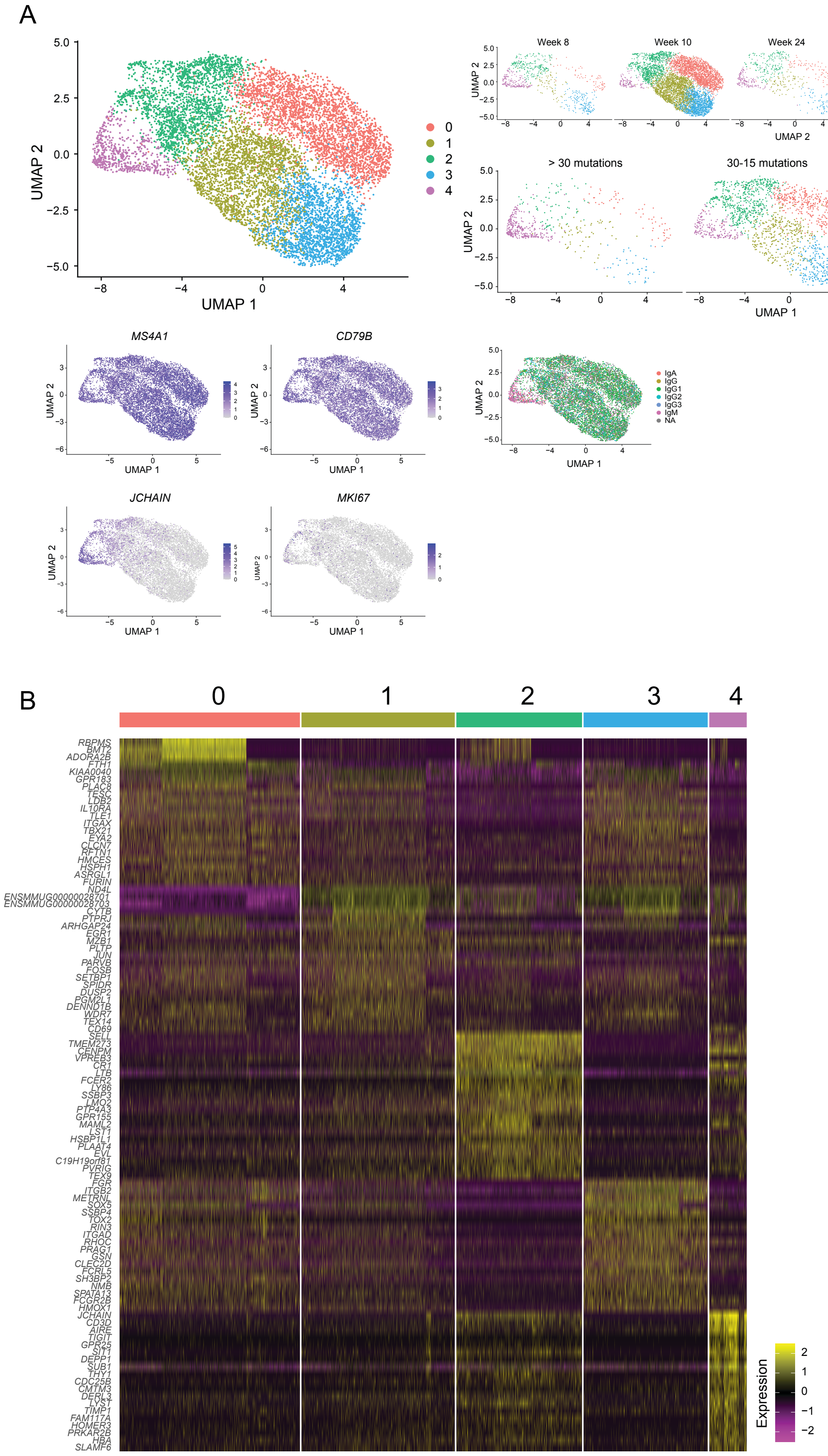
A
UMAP 1
UMAP 2
0
1
2
3
4
Week 8
Week 10
Week 24
Week 26
Week 32
> 30 mutations
30-15 mutations
0-14 mutations
MS4A1
CD79B
JCHAIN
MKI67
IgA
IgG
IgG1
IgG2
IgG3
IgM
NA
B
RBPMS
BMT2
ADORA2B
FTH1
KIAA0040
GPR183
PLAC8
TESC
LDB2
IL10RA
TLE1
ITGAX
TBX21
EYA2
CLCN7
RFTN1
HMCES
HSPH1
ASRGL1
FURIN
ND4L
ENSMMUG00000028701
ENSMMUG00000028703
CYTB
PTPRJ
ARHGAP24
EGR1
MZB1
PLTP
JUN
PARVB
FOSB
SETBP1
SPIDR
DUSP2
PGM2L1
DENND1B
WDR7
TEX14
CD69
SELL
TMEM273
CENPM
VPREB3
CR1
LTB
FCER2
LY86
SSBP3
LMO2
PTP4A3
GPR155
MAML2
LST1
HSBP1L1
PLAAT4
EVL
C19H19orf81
PVRIG
TEX9
FGR
ITGB2
METRNL
SOX5
SSBP4
TOX2
RIN3
ITGAD
RHOC
PRAG1
GSN
CLEC2D
FCRL5
SH3BP2
NMB
SPATA13
FCGR2B
HMOX1
JCHAIN
CD3D
AIRE
TIGIT
GPR25
SIT1
DEPP1
SUB1
THY1
CDC25B
CMTM3
DERL3
LYST
TIMP1
FAM117A
HOMER3
PRKAR2B
HBA
SLAMF6
Expression

Figure S7

Fig. S7. Transcriptomic analysis of Env-binding B_{Mem} cells in NHPs. (**A**) Uniform manifold approximation and projection (UMAP) visualization of single-cell gene expression profiles identifying clusters among Env-binding B_{Mem} cells sorted from PBMCs. Shown on the right are UMAP visualizations split by week (week 8, 10, 24, 26 and 32; top) or by the number of nucleotide heavy-chain mutations (more than 30 mutations, between 30 to 15 mutations or less than 15 mutations; middle). UMAP visualization depicting B cell isotypes in different colors is shown on the bottom right. Feature plots showing the expression of *MS4A1* (CD20), *CD79B*, *JCHAIN*, and *MKI67* are shown on the bottom left. (**B**) The heatmap shows the 20 most upregulated genes for each cluster.

Table S1. Rabbit study schema. All animals were immunized at weeks 0, 8, and 24. IM, intramuscular.

Group	N	Immunogen	mRNA/Protein	Dose (µg)	Route	Adjuvant
1	6	BG505 MD39.2 gp140	mRNA	100	IM	none
2	6	BG505 MD39.3 gp140	mRNA	100	IM	none
3	6	BG505 MD39.2 gp151	mRNA	100	IM	none
4	6	BG505 MD39.3 gp151	mRNA	100	IM	none
5	6	BG505 MD39.3 CD4KO gp151	mRNA	100	IM	none
6	6	BG505 gp120 foldon	Protein	30	IM	SMNP (375 µg)
7	6	BG505 MD39.3 soluble trimer	Protein	30	IM	SMNP (375 µg)
8	6	BG505 MD39 soluble trimer	Protein	30	IM	SMNP (375 µg)

Table S2. Flow Panel for MD39.3-binding B_{Mem} analysis

Antibodies	Clone	Source	Cat #	Dilution
Streptavidin BV711 (empty)	-	BioLegend	405241	-
Streptavidin AF647 (MD39.3)	-	BioLegend	405237	-
Streptavidin BV421 (MD39.3)	-	BioLegend	405225	-
Streptavidin PE (MD39.3 bKO)	-	BioLegend	405245	-
Fixable Viability Dye eFluor506	-	Thermo Fisher Scientific	65-0866-18	1:1000
Mouse anti-human CD4 BV650	OKT4	BioLegend	317436	1:100
Mouse anti-human CD8a APC-eFluor780	RPA-T8	Thermo Fisher Scientific	47-0088-42	1:100
Mouse anti-human CD16 APC-eFluor780	CB16	Thermo Fisher Scientific	47-0168-42	1:100
Mouse anti-human CD20 AF700	2H7	BioLegend	302322	1:100
Mouse anti-human IgG BUV737	G18-145	BD Biosciences	612819	1:100
Mouse anti-human CD27 PE-Cy7	O323	Thermo Fisher Scientific	25-0279-42	1:50
Mouse anti-human CD3 BUV395	SP34-2	BD Biosciences	564117	1:40
Goat anti-human IgD AF488	polyclonal	SouthernBiotech	2030-30	1:40
Mouse anti-human IgM PerCP-Cy5.5	G20-127	BD Biosciences	561285	1:40

Table S3. Flow Panel for MD39.3-binding B_{GC} analysis

Antibodies	Clone	Source	Cat #	Dilution
Streptavidin AF647 (MD39.3)	-	BioLegend	405237	-
Streptavidin BV421 (MD39.3)	-	BioLegend	405225	-
Streptavidin PE (MD39.3 bKO)	-	BioLegend	405245	-
Fixable Viability Dye eFluor506	-	Thermo Fisher Scientific	65-0866-18	1:1000
Mouse anti-human CD4 BV711	OKT4	BioLegend	317440	1:100
Mouse anti-human CD8a APC-eFluor780	RPA-T8	Thermo Fisher Scientific	47-0088-42	1:100
Mouse anti-human CD16 APC-eFluor780	CB16	Thermo Fisher Scientific	47-0168-42	1:100
Mouse anti-human CD20 AF488	2H7	BioLegend	302316	1:100
Mouse anti-human IgG BUV737	G18-145	BD Biosciences	612819	1:40
Mouse anti-human CXCR5 PE-Cy7	Mu5UBEE	Thermo Fisher Scientific	25-9185-42	1:20
Mouse anti-human CD3 BUV395	SP34-2	BD Biosciences	564117	1:40
Mouse anti-rhesus CD38 PE-Cy5	OKT10	Conjugated in house	-	1:100
Mouse anti-human IgM PerCP-Cy5.5	G20-127	BD Biosciences	561285	1:40
Mouse anti-human PD-1 BV605	EH12.2H7	BioLegend	329924	1:20
Mouse anti-human CD71 PE-CF594	L01.1	BD Biosciences	custom	1:20

Table S4. Flow Panel for sorting of MD39.3-binding B_{Mem} cells

Antibodies	Clone	Source	Cat #	Dilution
Streptavidin AF647 (MD39.3)	-	BioLegend	405237	-
TotalSeq-C Streptavidin BV421 (MD39.3)	-	BioLegend	custom	-
TotalSeq-C Streptavidin PE (MD39.3 bKO)	-	BioLegend	405155	-
Fixable Viability Dye eFluor506	-	Thermo Fisher Scientific	65-0866-18	1:1000
Mouse anti-human CD8a APC-eFluor780	RPA-T8	Thermo Fisher Scientific	47-0088-42	1:1000
Mouse anti-human CD16 APC-eFluor780	CB16	Thermo Fisher Scientific	47-0168-42	1:100
Mouse anti-human CD14 APC-Cy7	M5E2	BioLegend	301820	1:100
Mouse anti-human CD3 APC-Cy7	SP34-2	BD Biosciences	557757	1:100
Mouse anti-human CD20 BUV395	2H7	BD Biosciences	563781	1:100
Mouse anti-human IgG BV605	G18-145	BD Biosciences	563246	1:100
Mouse anti-human CD27 PE-Cy7	O323	Thermo Fisher Scientific	25-0279-42	1:50
Goat anti-human IgD AF488	polyclonal	SouthernBiotech	2030-30	1:50
Mouse anti-human IgM PerCP-Cy5.5	G20-127	BD Biosciences	561285	1:50

Table S5. Flow cytometry AIM and ICS panel staining for MD39.3-specific T cells

Antibodies	Clone	Source	Cat #	Dilution
LIVE/DEAD Fixable Blue	-	Thermo Fisher Scientific	L23105	1:500
GolgiPlug	-	BD Biosciences	555029	-
GolgiStop	-	BD Biosciences	554724	-
Mouse anti-human CD40	HB14	Miltenyi	130-094-133	1:200
Mouse anti-human CXCR5 PE-Cy7	MU5UBEE	Thermo Fisher Scientific	25-9185-42	1:100
Mouse anti-human CCR7 BV650	G043H7	BioLegend	353233	1:100
Mouse anti-human CD69 PE-Cy5	FN50	BioLegend	310908	1:250
Mouse anti-human CD137 (4-1BB) BV421	4B4-1	BioLegend	309819	1:250
Mouse anti-human CD25 BV605	BC96	BioLegend	302631	1:250
Mouse anti-human CD40L BB515	24-31	BD Biosciences	568170	1:250
Mouse anti-human CD134 (OX40) PE	L106	BD Biosciences	340420	1:250
Mouse anti-human CD8 BUV496	RPA-T8	BD Biosciences	612943	1:100
Mouse anti-human CD14 APC-Cy7	M5E2	BioLegend	301820	1:100
Mouse anti-human CD16 APC-eFluor780	eBioCB16	Thermo Fisher Scientific	47-0168-42	1:100
Mouse anti-human CD20 APC-Cy7	2H7	BioLegend	302314	1:100
Mouse anti-human CD3 BUV395	SP34-2	BD Biosciences	564117	1:100
Mouse anti-human CD4 PerCP-Cy5.5	OKT4	BioLegend	317428	1:100
Mouse anti-human PD-1 BV785	EH12.2H7	BioLegend	329929	1:100
Mouse anti-human CD45RA PE-CF594	5H9	BD Biosciences	565419	1:100
Armenian Hamster anti-ICOS BV480	C398.4A	BD Biosciences	566087	1:100

Mouse anti-human IFN-γ BUV737	4S.B3	BD Biosciences	612845	1:100
Rat anti-human IL-2 BV750	MQ1-17H12	BD Biosciences	566361	1:200
Mouse anti-human TNF-α BV711	MAb11	BioLegend	502940	1:200
Mouse anti-human Granzyme B Alexa Fluor 700	GB11	BD Biosciences	560213	1:1000
Mouse anti-human IL-21 Alexa Fluor 647	3A3-N2.1	BD Biosciences	560493	1:200
Human Fc Block	Fc1	BD Biosciences	564220	-

Table S6. Primer sequences used for Rhesus macaque scBCR-seq library preparation

	Target region	PCR	Sequence
V2_10X_forward_Bmix1	-	Outer/Inner	GATCTACACTCTTTCCCTACACGACGC
IGHA_outer	IGHA	Outer	GGCTGGGATTTGTGTAGTGC
IGHM_outer	IGHM	Outer	CTCTCAGGACTGATGGGAAGC
IGHG_outer	IGHG	Outer	TTGTCCACCTTGGTGTTGCT
IGLC_outer	IGLC	Outer	GTCTCCACTCCCGCGTTGAC
IGKC_outer	IGKC	Outer	AGGTGTTGTCCTTGCTGTCC
IGHA_inner	IGHA	Inner	TCACGTTGAGTGGCTCCT
IGHM_inner	IGHM	Inner	AACGGCCACTTCGTTTGT
IGHG_inner	IGHG	Inner	AGCCCTGAGGACTGTAGGA
IGLC_inner	IGLC	Inner	ATCTGCCTTCCAGGCCA
IGKC_inner	IGKC	Inner	ACCTTCCACTTTACGCT

Data file S1. Individual-level data for experiments where n<20.
